# Galectin-3/CD146 interaction promotes renal damage and systemic inflammation after acute kidney injury

**DOI:** 10.1038/s41598-025-25477-4

**Published:** 2025-11-24

**Authors:** L. Boutin, S. M. Figueroa, E. Roger, S. Hadjadj, R. Piedagnel, E. Gayat, J.-L. Samuel, M. Legrand, F. Dépret, Christos E. Chadjichristos

**Affiliations:** 1 Department of Anesthesia and Critical Care, Hôpital Européen Georges Pompidou (HEGP), Leblanc, Paris, France; 2 INSERM, UMR-942, MASCOT, Cardiovascular Markers in Stress Conditions, Université de Paris Cité, Paris, 75010 France; 3 INSERM, UMR-S1155, Bâtiment Recherche, Tenon Hospital, Faculty of Medicine, Sorbonne University, Paris, 75020 France; 4 Division of Critical Care Medicine, UCSF, 8785, Anesthesia and Peri-operative Care, San Francisco, CA 8785 USA

**Keywords:** Galectin-3, CD146, AKI, Renal ischemia-reperfusion, Systemic inflammation, Nephrology, Acute kidney injury

## Abstract

Galectin-3 (Gal-3) expression is abnormally increased after renal ischemic injury, in various studies, further promoting tissue damage. Gal-3 may interact with the endothelial layer to induce inflammation during renal injury, linking the systemic and tissue compartments. However, this mechanism has not been investigated after acute kidney injury (AKI). To this end, wild-type (WT) and Gal-3-/- mice underwent renal ischemia followed by reperfusion (rIR). Bone marrow transplantation was used to differentiate Gal-3 expression in renal tissue from systemic bone marrow cells. WT mice were injected with recombinant Gal-3 to study systemic issues. Gal-3 expression was assessed by immunofluorescence, western blot, and scRNAseq. Systemic pathways were analyzed with plasma proteomics. Immunofluorescence and scRNAseq showed increased Gal-3 expression after rIR, in different tubular segments and infiltrating immune cells within damaged kidneys. In bone marrow transplanted mice, renal dysfunction, damage, and inflammation were higher when Gal-3 was expressed in renal tissue. Gal-3 upregulation was associated with endothelial activation and renal inflammation after rIR, both of which were prevented in Gal-3-/- mice. Similar results were confirmed by plasma proteomics, as these mice showed reduced endothelial damage and rIR-associated inflammatory pathways. Systemic injection of recombinant Gal-3 increased the endothelial adhesion molecule CD146 expression in the kidney, without promoting renal injury. Interestingly, isolated renal tubules showed increased Gal-3 levels, interacting with CD146 to promote cytokine secretion and immune cell infiltration in the kidney. Thus, Gal-3 may promote endothelial activation via direct interaction with the endothelial activation marker CD146 after AKI, leading to renal injury and further systemic inflammation.

**Supplementary Information:**

The online version contains supplementary material available at 10.1038/s41598-025-25477-4.

## Introduction

Acute kidney injury (AKI) is associated with high mortality and morbidities^1^. Consequently, AKI leads to systemic inflammation and endothelial dysfunction through complex pathophysiological processes^2–5^. We have recently identified Galectin-3 (Gal-3) as a key player in the progression of systemic pathways after renal ischemia-reperfusion in the context of type 3 cardiorenal syndrome (AKI leading to acute heart failure)^6,7^. In addition, we and others have reported that elevated Gal-3 levels are associated with AKI, renal dysfunction^6,8–10^, and suggested a link between AKI and systemic inflammation^11,12^.

Gal-3 is a member of the lectin family that binds to β-galactosidase and is expressed in various tissues and organs^6,13,14^. It is involved in various physiopathological processes such as development, cell differentiation, migration, inflammation, and fibrosis. Gal-3 expression can occur either inside the cell, on the cell membrane, or in the plasma. In its soluble form, Gal-3 can act as a damage-associated molecular pattern (DAMP) and is secreted in vesicles by injured cells to promote monocyte infiltration and further fibrosis within the damaged tissues^8,15^. Gal-3 is also expressed by immune cells such as macrophages or lymphocytes, underlying its importance in the systemic consequences following AKI^6^. Furthermore, Gal-3 is also known to be upregulated in the kidney after renal injury, strengthening the hypothesis that this lectin may be related to the relationship between AKI and systemic inflammation^13,16^. We have previously reported that Gal-3 originating from bone marrow-derived cells, had a key role in remote cardiac injury via a systemic pathway^6^. Thus, we proposed a systemic role for Gal-3 in the cardiorenal axis through immune cell infiltration. Gal-3 may interact with the endothelial layer, linking the systemic and the tissue compartments, thereby promoting systemic inflammation and distant organ dysfunction. However, this issue is currently understudied. Such a study can provide valuable insights into interactions involved in renal systemic pathways following AKI. To this end, in the current study, we focused on the role of Gal-3 in AKI after renal ischemia/reperfusion, and we investigated the interaction of tissue Gal-3 in the kidney with the vascular endothelium and its systemic consequences after renal injury. We demonstrated that Gal-3 produced by renal tubular cells after AKI, is involved in the progression of inflammation and that this event implies an interaction between Gal-3 and the endothelial dysfunction marker CD146.

## Results

### Gal-3 expression is increased mainly in tubular cells after rIR

**Fig. 1:**
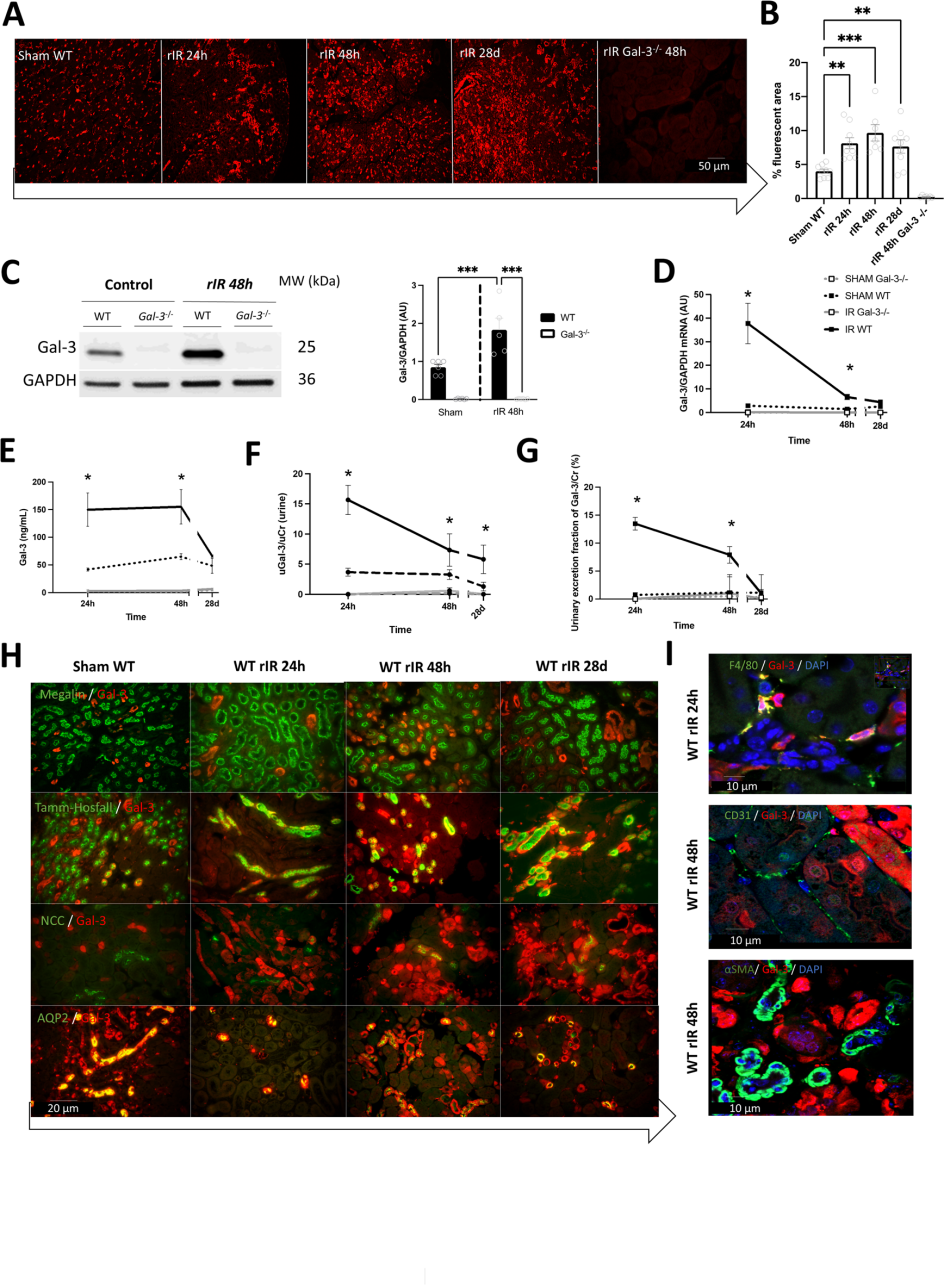
Gal-3 expression is increased 24 h after rIR and exhibits multicellular colocalization. Immunofluorescence (**A**) and quantification (**B**) of Gal-3 from 24 h to 28d post-rIR, and Western blot analysis and quantification (**C**) at 48 h post-rIR. Gal-3 mRNA expression (**D**) was assessed from 24 h to 28d post-rIR, and Gal-3 levels in plasma (**E**), urine (**F**), and calculated Gal-3 urinary excretion fraction according to creatinine (**G**), were measured over the same period. Representative images from colocalization of Gal-3 with tubular markers (**H**), such as proximal tubule (megalin), Henle’s loop (Tamm-Horsfall), distal tubule (Na+-Cl − cotransporter, NCC), collecting duct (aquaporin 2, AQP2), and immune cell markers (**I**) (macrophage F4-80, endothelial CD31 and myofibroblast αSMA), in damaged kidneys, from 24 h to 28d post-rIR in WT mice. Data are presented as mean ± SEM (*n* = 4–12). Two-way ANOVA corrected with Bonferroni transformation; *P < 0.05, **P < 0.01, ***P < 0.001.

To assess Gal-3 expression within injured kidneys after rIR we performed immunofluorescence, western blot, and qPCR experiments in WT and Gal-3-/- mice. We confirmed a pronounced increase in Gal-3 at both protein and mRNA levels in damaged kidneys of WT mice, extending from 24 h to 28d after rIR (Fig. 1A-D**)**. As expected, Gal-3 was not detectable in Gal-3-/- mice. In addition, Gal-3 expression was significantly increased in the plasma and urines of WT mice at 24 h, followed by a subsequent decrease by 28d (Fig. 1E-F). To distinguish between AKI-induced Gal-3 accumulation and Gal-3 release, we corrected the urinary excretion fraction with creatinine, suggesting that Gal-3 excretion was increased under pathological conditions (Fig. 1G).

To identify the precise cellular localization of Gal-3 expression, we performed colocalization experiments with different tubular cell markers, such as megalin (proximal tubules), Tamm-Horsfall (Henle’s loops), Aquaporin-2 (AQP2, collecting ducts), and NCC (distal tubules) (Fig. 1H). Interestingly, from 24 h to 28d after rIR, Gal-3 expression predominantly colocalized with markers of proximal and collecting ducts and seems to expand to Henle’s loop and proximal tubule 48 h after rIR and return to basal expression at 28d. Furthermore, we confirmed Gal-3 colocalization with F4-80 (macrophages)^6^. However, no colocalization was observed with the endothelial marker CD31/PECAM or the smooth muscle cell marker αSMA after rIR (Fig. 1I).

To confirm our above-described observations, several analyses were performed based on the Humphrey lab scRNAseq dataset^17–21^. In healthy mice, Gal-3 was mainly expressed in the collecting (CD-PC) and connecting ducts (CNT)^19^ (Figure S1A). In pathological conditions, Gal-3 expression was spread to other tubular cells, such as intercalated cells (IC) and proliferative proximal tubules (prolif. PT) (Figure S1B)^19^. After rIR, from 4 to 12 h, Gal-3 expression was increased in tubules, including principal cells (PC1, PC2), connecting and distal convoluted tubules (CNT & DCT), macula densa cells (MD), and cortical thick ascending limb (CTAL). Interestingly, at 12 h, Gal-3 expression was also increased in macrophages (Mϕ), T cells, and endothelial cells (EC1 & EC2) (Figure S1C)^20^. Finally, this widespread expression of Gal3 was confirmed by spatial transcriptomics in mice following rIR from 4 h to 6 weeks (Figure S1D)^21^.

### Gal-3 deletion protects renal function and structure in mice after AKI

**Fig. 2:**
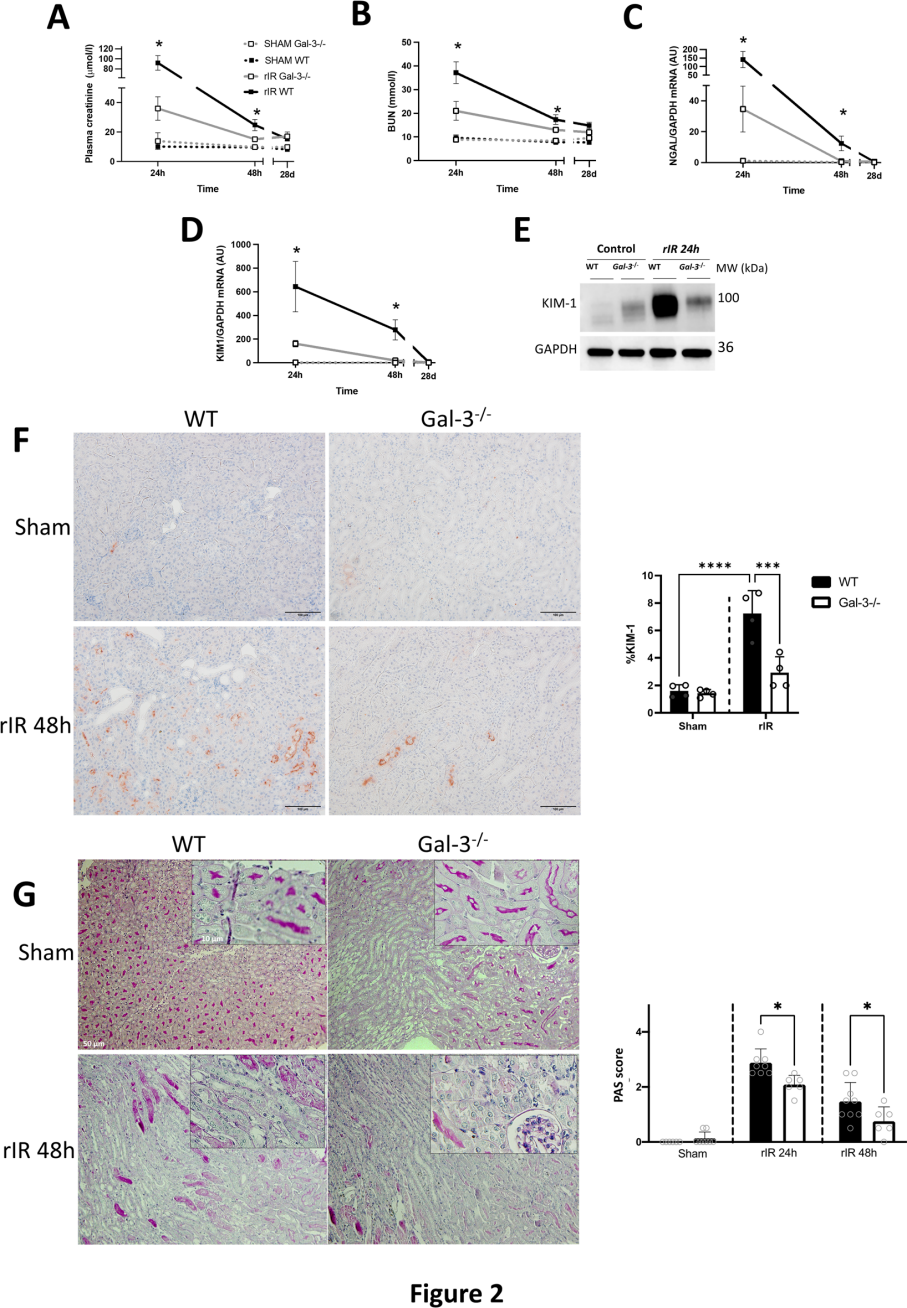
Renal function and structure in WT and Gal-3-/- mice after rIR. Different indicators were examined to evaluate kidney damage. Plasma creatinine (**A**) and BUN levels (**B**) were measured for kidney function, and NGAL (**C**) and KIM-1 (**D**) mRNA expression for renal damage. KIM-1 protein expression (**E**) was assessed 24 h post-rIR. KIM-1 immunostaining (F) and PAS staining (G) to evaluate kidney damage were performed after 48 h of rIR. Data are presented as mean ± SEM (*n* = 4–12). Two-way ANOVA corrected with Bonferroni transformation; *P < 0.05, **P < 0.01, and ***P < 0.001.

To study the impact of the upregulation of Gal-3 in the kidney, we asses renal function and structural damage in WT and Gal3-/- mice from 24 h until 28 days after rIR. Gal-3-/- mice showed preserved renal function throughout this period since plasma creatinine and blood urea nitrogen (BUN) significantly improved (Fig. 2A-B). Similar results were observed for the mRNA expression of renal injury markers such as Neutrophil gelatinase-associated lipocalin (NGAL) and Kidney injury molecule-1 (KIM-1). Indeed, there was a significant upregulation of these markers after rIR compared to sham mice, and their expression returned to baseline levels by day 28. Importantly, this increased expression was blunted in Gal-3-/- mice (Fig. 2C-F). In accordance, histological analysis using PAS staining revealed a reduction in tubular damage in Gal-3-/- mice subjected to rIR (Fig. 2G). These data suggest that genetic deletion of Gal-3 improves renal function and structure in mice after rIR.

### Gal-3 increased expression is associated with renal and systemic inflammation after AKI

**Fig. 3:**
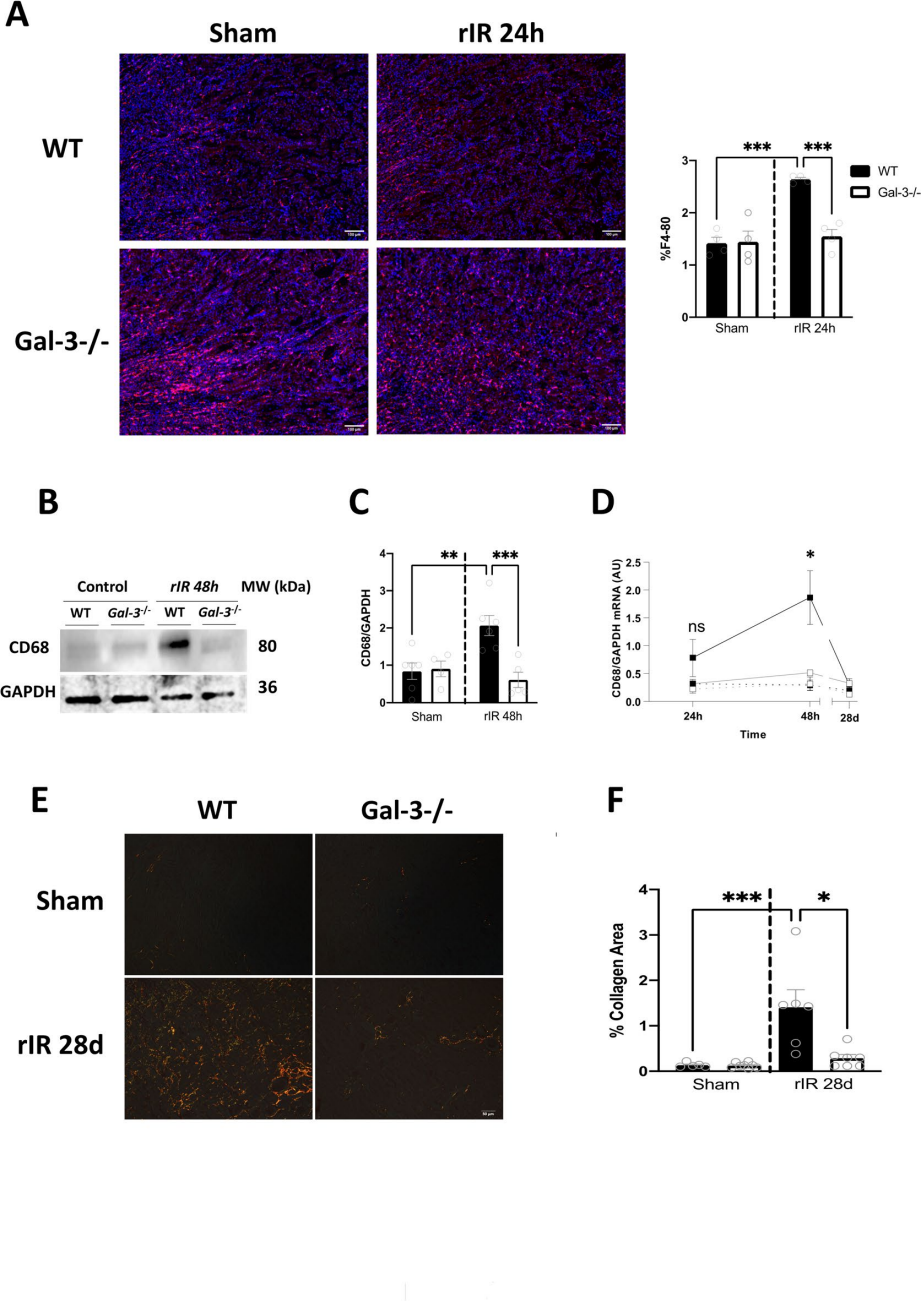
Gal-3 increased expression was associated with renal inflammation. Immunostaining of F4-80 in renal tissue 24 h after rIR (**A**) and quantification. CD68 Western blot analysis (**B**) and quantification (**C**), and mRNA expression (**D**) in kidneys after 48 h of rIR. Sirius Red immunostainings (**E**) and its quantification (**F**) were performed after 28 days of rIR. Data are presented as mean ± SEM (*n* = 4–12). Two-way ANOVA corrected with Bonferroni transformation; **P < 0.01 and ***P < 0.001.

Gal-3 increased expression has been associated with renal inflammation. To further confirm this relationship, we performed immunostainings for F4-80 on kidney sections at 24 h after rIR. We observed an increased monocyte infiltration in WT kidneys and a decrease in Gal-3-/- injured ones (Fig. 3A). In accordance, CD68 mRNA and protein expression increase was blunted in Gal-3-/- mice at 48 h post-rIR (Fig. 3B-D). Furthermore, at 48 h post-IR, Gal-3 seems to promote a M1 macrophage phenotype since CD80 mRNA increased levels were significantly decreased in Gal-3-/- mice **(Figure S2**). The renoprotective effect of Gal-3 genetic deletion was confirmed by Sirius Red staining, which demonstrated reduced renal fibrosis in Gal-3-/- mice 28 days after rIR (Fig. 3E-F). Thus, our findings suggest that genetic inhibition of Gal-3 confers significant protection against renal dysfunction and preserves both the structural and functional integrity of the kidneys following rIR.

**Fig. 4:**
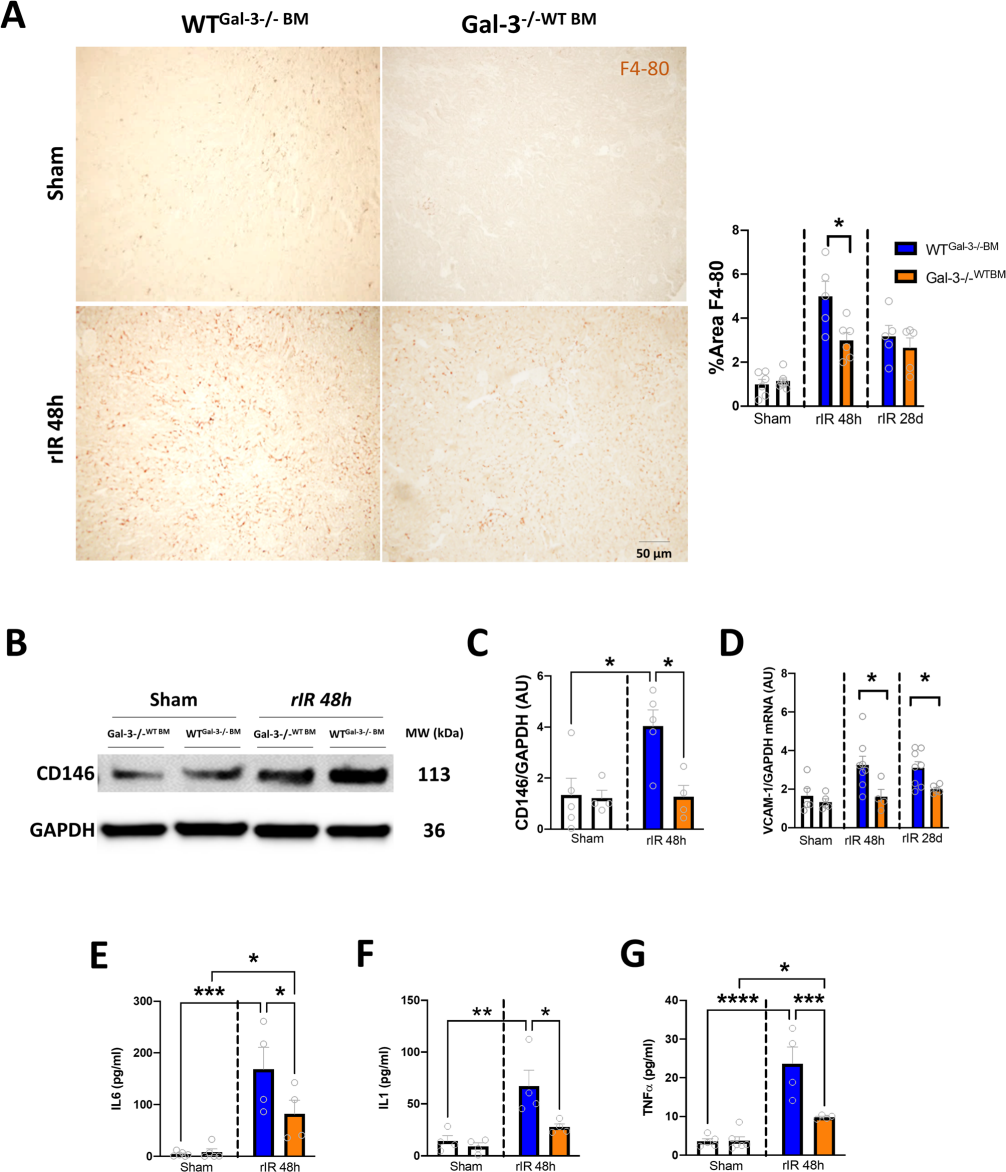
Tissue Gal-3 increased expression was associated with kidney and systemic inflammation after rIR. Adoptive transfer of bone-marrow-derived cells from WT or Gal-3-/- mice, obtained WTGal-3-/-BM (Gal-3 present only in renal tissue) and Gal-3-/-WTBM (Gal-3 present in bone marrow-derived cells). Immunostaining for F4-80 and quantification in damaged kidneys after 48 h of rIR (**A**). WB for CD146 (**B**) and quantification (**C**), VCAM-1 mRNA expressions (**D**). Plasma levels of IL-6 (**E**), IL-1 (**F**), and TNFα (**G**) after 48 h of rIR. Data are presented as mean ± SEM (*n* = 3–12). Two-way ANOVA corrected with Bonferroni transformation; *P < 0.05, **P < 0.01, ***P < 0.001 and ****P < 0.0001.

We have previously reported and confirmed that Gal-3 increased expression was detected in both immune and renal tubular cells. To distinguish between Gal-3 expression in renal tissue and that derived from immune cells, we established a model of irradiated and bone marrow-transplanted mice, resulting in two distinct groups: WT^Gal−3−/−BM^ (expressing Gal-3 only in renal tissue) and Gal-3-/-^WTBM^ (expressing Gal-3 only in bone marrow-derived cells). After rIR, WT^Gal−3−/−BM^ showed significantly more renal infiltrates, as illustrated by F4-80 immunostaining (Fig. 4A). The inflammatory process was associated with increased endothelial dysfunction marker CD146 and Vascular cell adhesion marker 1(VCAM1) mRNA levels and CD146 protein expression, compared to sham mice, and this effect was significantly more pronounced in WT^Gal−3−/−BM^ mice, 48 h and 28d after rIR (Fig. 4B–D). Consistent with these findings, measurements of plasma cytokines, including Interleukin-1 (IL1), Interleukin-6 (IL6), and Tumor Necrosis Factor α (TNFα), were significantly higher in WT^Gal−3−/−BM^ after 48 h of rIR (Fig. 4E–G). These data were confirmed by plasma proteomic analysis, showing in a comparison between WT^Gal−3−/−BM^ and Gal-3-/-^WTBM^ mice, that the cytokine and chemokine processes were predominantly upregulated when Gal-3 was expressed only within the renal tissue. This included plasma proteins such as IL-6, Chemokine (C-C motif) Ligand 5 (CCL5), IL-10, CXC motif chemokine Ligand 9 (CXCL9), IL-17, CXCL1, and CCL20 (Figure S3).

The inflammatory response was strongly correlated with renal dysfunction. After 48 h of rIR, WT^Gal−3−/−BM^ mice had significantly higher plasma creatinine and BUN levels (Figure S4A-B). There was also a significant increase in the mRNA expression of renal damage markers NGAL and KIM-1 in WT^Gal−3−/−BM^ mice compared to Gal-3-/-^WTBM^ mice (Figure S4C-D). In addition, renal damage was evident, as indicated by the PAS histologic score, which was significantly higher in WT^Gal−3−/−BM^ mice (Figure S4E-F). As shown in Fig. 1, after 48 h of rIR, Gal-3 was increased at both the mRNA and protein levels. Interestingly, this increased expression was significantly more pronounced in the renal tissue (WT^Gal−3−/−BM^) compared to Gal-3-/- kidneys injected with BM cells from WT mice (Gal-3-/-^WTBM^). The difference in Gal-3 levels was also observed in the plasma, indicating a significantly higher level of Gal-3, when it was only expressed by damaged tissues. This suggests potential release or secretion of Gal-3 (Figure S5A). Interestingly, this Gal-3 increased expression in WT^Gal−3−/−BM^ appeared to be mainly localized in the renal tubules, as shown in immunofluorescence images (Figure S5B-C). Furthermore, both Gal-3 mRNA and protein levels increased in renal tissue (Figure S5D-F). These findings suggest that the increased expression of Gal-3 within the damaged kidney tissue plays a pivotal role in AKI and the subsequent systemic response following rIR.

### Gal-3 increased expression is associated with endothelial activation and proinflammatory pathways and promotes CD146 expression post-rIR

To gain deeper insights into the pathophysiological pathways involving Gal-3 in AKI, we performed a plasma proteomic analysis in both WT and Gal-3-/- mice after 48 h of rIR. This analysis allowed us to identify a panel of proteins and assess their relative changes, as shown in Figure S6, using Log2fold change. When comparing WT sham mice with WT mice after 48 h of rIR, the most significantly expressed proteins were associated with apoptosis, cell adhesion, and cytokine pathways. When Gal-3-/- mice were compared 48 h after rIR with WT mice, the most significantly increased proteins in WT mice included those related to cell signalling and adhesion (e.g., cadherin-6 and EPCAM) and some cytokines/chemokines (CCL5, IL-10, and CXCL9) (Figure S6). These results suggest that cell adhesion-induced signalling, together with chemokine and cytokine mechanisms, may play an important role in the renal pathophysiology associated with increased Gal-3 expression after rIR.

**Fig. 5:**
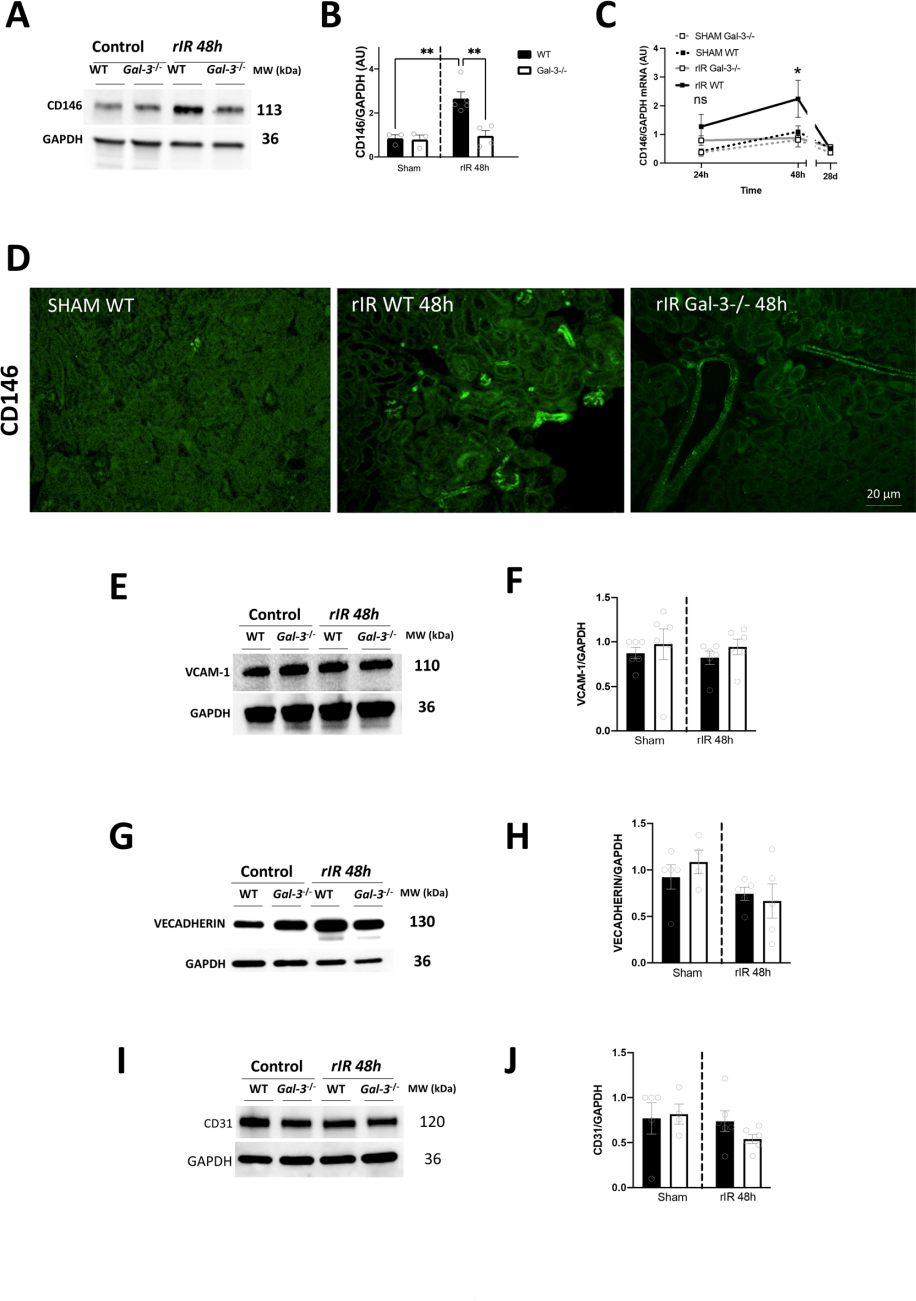
CD146 increased expression 48 h after rIR is Gal-3 dependent. Kidneys from WT and Gal-3-/- mice were assessed following rIR injury. CD146 Western Blot (**A**) and quantification at 48 h post-rIR (**B**), CD146 mRNA expression from 24 h to 28d post-rIR (**C**), and immunofluorescence 48 h post-rIR (**D**). Protein expression of VCAM-1 (**E**) and its quantification (**F**), VE-cadherin (**G**) and its quantification (**H**), and CD31 (**I**) and its quantification (**J**) 48 h post-rIR. Data are presented as mean ± SEM (*n* = 4–12). Two-way ANOVA corrected with Bonferroni transformation; *P < 0.05 and **P < 0.01.

To gain insights into how Gal-3 influences inflammation and injury in the context of rIR, we evaluated markers of endothelial activation. At 48 h post-rIR, we observed an increase in the renal expression of the endothelial dysfunction marker CD146 in WT mice, at both the mRNA and protein levels (Fig. 5A-C). Immunofluorescence of CD146 further revealed increased expression, mainly localized in peritubular and glomerular endothelial cells following rIR (Fig. 5D). Surprisingly, we did not observe any significant changes in other endothelial markers, such as VCAM-1, Vascular endothelial cadherin (VE-Cadherin) and CD31, in WT compared to Gal-3-/- mice, within injured kidneys after 48 h of rIR (Fig. 5E-J). These results suggest a selective upregulation of CD146 expression, independently of endothelial activation.

### Injection of recombinant Gal-3 enhances CD146 expression and cytokine secretion independently of renal damage

**Fig. 6:**
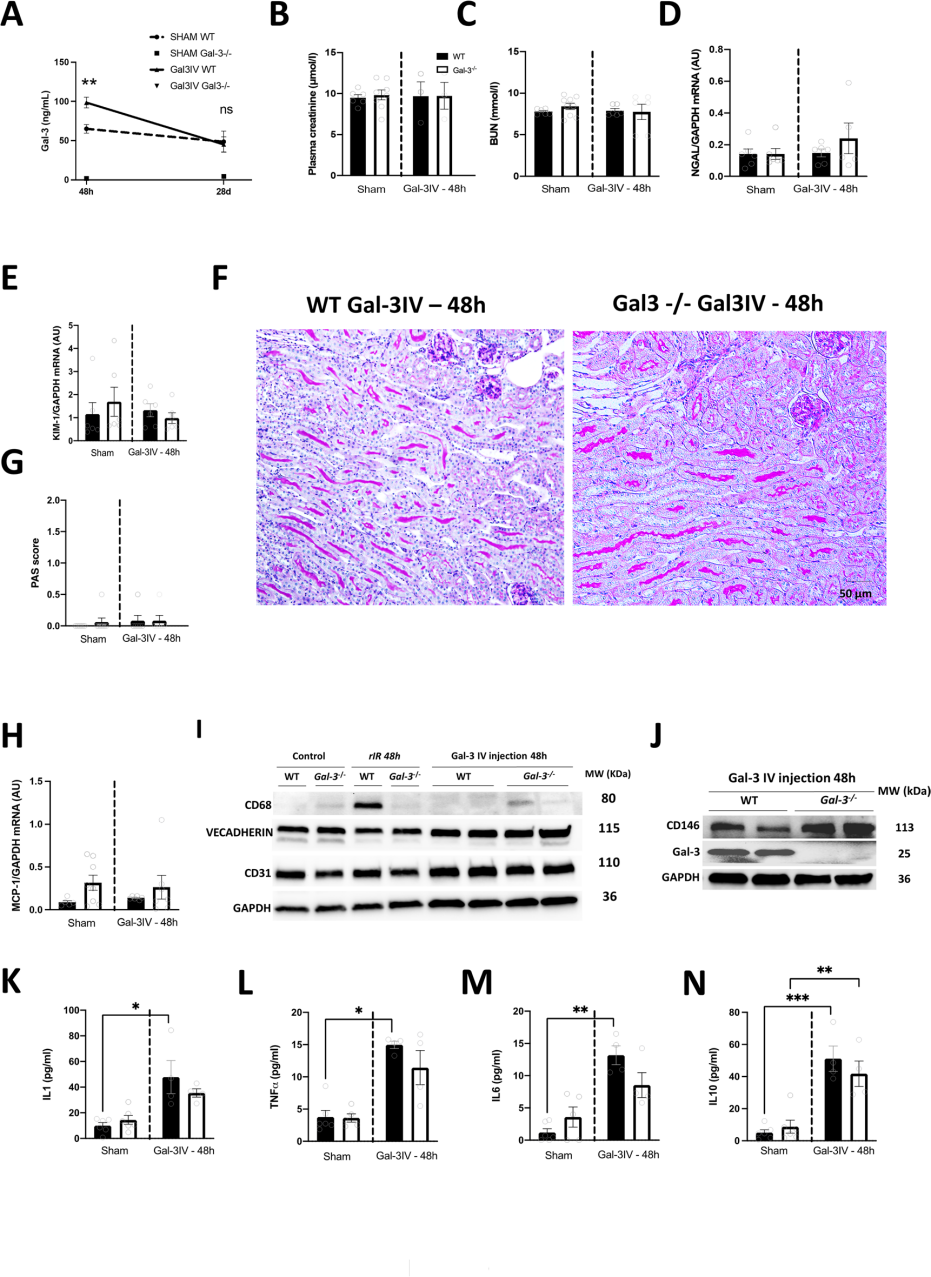
Pro-inflammatory mediators are increased by recombinant Gal-3 injection, regardless of renal dysfunction. 48 h after Gal-3 injection in WT and Gal-3-/- mice, plasma Gal-3 (**A**), plasma creatinine (**B**), and BUN (**C**) levels were measured. Kidney mRNA expression of NGAL (**D**) and KIM-1 (**E**), kidney histological analyses with PAS staining (**F**), and damage scoring (**G**), were assessed. After 48 h of rIR, kidney mRNA expression of MCP-1 (**H**) and Western Blot for CD68, VECADHERIN, CD31 (**I**), and CD146, and Gal-3 (**J**) were also assessed. We further analyzed plasma levels for inflammatory markers such as IL-1 (**K**), TNFα (**L**), IL-6 (**M**), and IL-10 (**N**). Data are presented as mean ± SEM (*n* = 4–6). Two-way ANOVA corrected with Bonferroni transformation; *P < 0.05, **P < 0.01, and ***P < 0.001. Dark histograms correspond to WT and open ones to Gal-3-/-.

To determine whether elevated plasma levels of Gal-3 could promote systemic inflammation, we administered recombinant Gal-3 to both WT and Gal-3-/-mice, without inducing rIR injury (Fig. 6A). Intravenous injection of recombinant Gal-3 did not induce renal dysfunction in mice, as no apparent changes in plasma creatinine and BUN levels were observed (Fig. 6B,C). Similarly, there was no increase in biomarkers of renal tubular damage at the mRNA levels, such as NGAL and KIM-1, or in the protein levels of KIM-1 (Fig. 6D,E; Figure S7A). In accordance, we did not observe any histological kidney damage at 48 h after the administration of recombinant Gal-3, as evidenced by PAS staining (Fig. 6F-G). Furthermore, Gal-3 injection did not promote renal immune cell infiltration, as indicated by the lack of significant differences between sham and Gal-3-injected mice in Monocyte chemoattractant protein-1 (MCP-1) mRNA and CD68 protein or mRNA expressions (Fig. 6H,I; Figure S7). Interestingly, the systemic injection of recombinant Gal-3 did not lead to an increase in Gal-3 expression in the kidney at 48 h in both WT and Gal-3-/- mice (Fig. 6J; Figure S7). However, the injection of Gal-3 resulted increased expression of CD146 in the renal tissue, at both the mRNA and protein levels, independently of the activation of endothelial markers such as CD31, VE-cadherin, and VCAM-1(Fig. 6I,J; Figure S7). Furthermore, injection of recombinant Gal-3 was associated with an increase in plasma proinflammatory cytokines, including IL1, IL6, TNF-α, and IL10 at 48 h after injection in both Gal-3-/- and WT mice compared with sham-operated mice (Fig. 6K-N). These results suggest that the injection of recombinant Gal-3 can induce a systemic inflammatory response and modulation of the endothelial CD146 marker, independently of tissue inflammation.

### Gal-3 interacts with CD146 after rIR

**Fig. 7:**
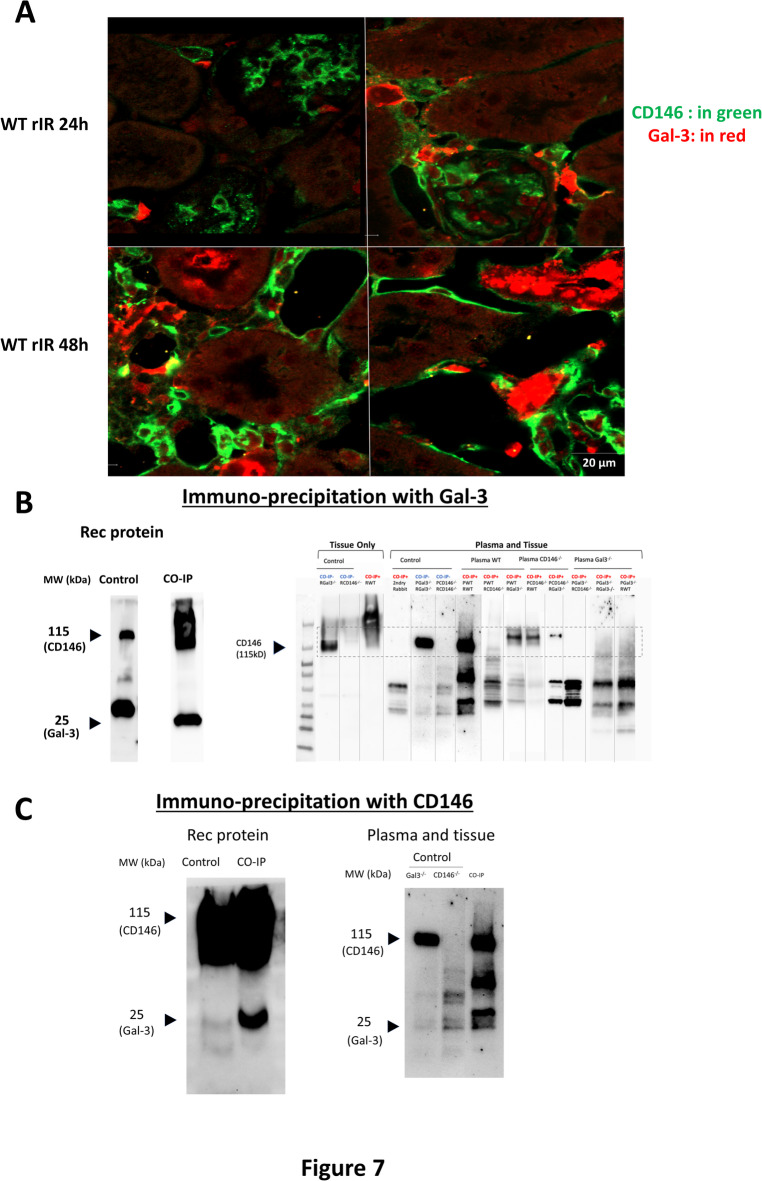
Gal-3 interacts with CD146. Representative immunostainings in the kidney of CD146 (in green) and Gal3 (in red) after 48 h of rIR (**A**). Immunoprecipitation of Gal3 and revelation of CO-IP for Gal3 and CD146 using recombinant (rec) protein with tissue and tubular lysates (**B**). Immunoprecipitation of CD146 and revelation of CO-IP for Gal3 and CD146 using recombinant protein with tissue and tubular lysates (**C**).

After confirming the potential role of Gal-3 in renal tissue after rIR, we investigated its interaction with endothelial markers. As reported above, Gal-3 appeared to affect CD146 expression and cytokine secretion after rIR. In addition, a recent in vitro study suggested CD146 as a Gal-3 receptor^22^. We thus hypothesized that Gal-3 secreted by tubular cells could interact with the endothelial CD146. RNA extraction from isolated tubular cells from WT mice after 48 h of rIR, confirmed increased expression of Gal-3 together with tubular damaged markers, such as KIM-1 and NGAL, as well as the stress-induced transcription factor Hypoxia-inducible factor 1α (HIF-1α) (Figure S8). This increased expression was blunted in Gal-3 KO tubules. Interestingly, Gal-3 was also measured in supernatants of isolated tubules cultured for 48 h in an appropriate medium after rIR. Similar results were observed in the supernatant of the MDCK tubular cell line under stress conditions (upon LPS stimulation). These data suggest that renal tubular cells under stress can produce and secrete Gal-3. To further verify a possible interaction between Gal-3 and CD146, we examined their colocalization 48 h after rIR by immunofluorescence with confocal microscopy. While there was no obvious colocalization between CD146 and Gal-3, these proteins were tightly localized in peritubular capillaries and tubular cells, respectively (Fig. 7A). Furthermore, CO-IP experiments with recombinant proteins confirmed the interaction between CD146 and Gal-3. Interestingly, this interaction was evident when using a combination of whole kidney tissue lysate and plasma from WT mice or CD146-/- mice, but not when using plasma from Gal-3-/- mice (Fig. 7B-C). These results suggest that the interaction between Gal-3 and CD146 is facilitated by the presence of a systemic ligand (Gal-3) and a tissue-bound receptor (CD146).

## Discussion

We have previously reported that Gal-3-dependent pathways promote remote cardiac injury after AKI in mice. In this study, we focused on the role of this lectin in renal injury and function after rIR. We provide evidence that Gal-3, produced by tubular damaged cells, was upregulated in renal tissue and secreted into the plasma. Gal-3 secretion was associated with endothelial-dependent mechanisms involving CD146, which further led to the secretion of chemokines and cytokines promoting tissue injury and fibrosis.

In our study we show increased expression of Gal-3 in the injured renal tissue, in both renal tubular and immune cells after renal IR. We observed a discrepancy between Gal-3 mRNA and protein expression levels, with mRNA peaking at 24 h, while protein levels reached their maximum at 48 h. This delay has been previously reported in the literature and is attributed to post-transcriptional regulation, specifically the stabilization of Gal-3 mRNA^6,23^. This upregulation is likely attributable to several pathophysiological processes activated during the renal repair phase. Indeed, according to the literature after renal IR, the proximal tubule epithelium undergoes necrosis and apoptosis, followed by a regenerative response involving dedifferentiation, proliferation, and migration of surviving tubular cells. Gal-3 is involved in orchestrating this repair response by modulating cell-cell and cell-matrix interactions^24^. In addition, Gal-3 increased expression in proliferating proximal tubules reflects its active role in promoting epithelial regeneration and restoration of tubular architecture^25^. However, Gal-3 plays a dual role by promoting profibrotic responses. In the early phase post-injury, Gal-3 may limit inflammation and support clearance of apoptotic cells. Persistent or dysregulated Gal-3 expression can lead to maladaptive repair, driving interstitial fibrosis and chronic kidney disease progression by activating fibroblasts and modulating TGF-β signaling^24^. Thus, the marked increase of Gal-3 in proliferative proximal tubules post IR highlights its central role in the delicate balance between regeneration and fibrosis. This supports its value as both a biomarker of injury and repair and a potential therapeutic target to modulate outcomes after AKI.

In the clinics, AKI is associated with a poor prognosis due to systemic consequences^26,27^. Therefore, identifying the mechanisms that trigger AKI and its consequences could improve the outcome of patients suffering from the disease^27^. Consistent with our study, increased expression of Gal-3 in the kidney was associated with tissue inflammation, fibrosis, and alterations in renal function in murine models of experimental AKI. Indeed, inhibition of Gal-3 by modified citrus pectin (MCP) improved renal function and tubulointerstitial injury after rIR and hypertension-induced nephropathy in rats^28,29^. Similar results were observed in cisplatin-induced and sepsis-associated AKI in mice^30,31^. Furthermore, a recent study highlighted the importance of the Gal-3 released by damaged renal tubular cells during folic acid-induced AKI, in renal inflammation and tissue damage. Indeed, interaction of Gal-3 with platelet glycoprotein VI enhanced interactions between monocytes and platelets, leading to the formation of monocyte-platelet aggregates and further the polarization of M1 macrophages as it seems to be the case in our study, at least after 48 h of renal IR^32^. In addition, our plasma proteomic studies and RNAseq dataset (http://humpreyslab.com/SingleCell/) have identified an association between Gal-3 and tissue damage in the kidneys of mice exposed to different models of experimental AKI or in humans (Figures S1 and S9). Interestingly, we have recently reported that elevated plasma levels of Gal-3 were strongly associated with renal dysfunction and an increased risk of major adverse renal events and further death in patients after admission to the intensive care units^9^. Another study also reported that serum concentrations of Gal-3 predicted AKI in patients with sepsis^31^. Furthermore, single-cell transcriptomics reported increased Gal-3 expression in the kidneys of patients with kidney allograft transplantation^33^. Thus, animal and clinical studies support the deleterious role of Gal-3 increased expression in organ pathophysiology after renal injury^8–10^. In this context, our work strengthens the hypothesis of Gal-3 as a primary trigger of systemic consequences but also underlines its importance in this complex interplay between kidneys and other organs^16,34^.

Few studies have focused on the systemic and inflammatory interactions between the kidneys and other organs^34^. We and others have previously reported that Gal-3, associated with immune cells, promotes cardiac fibrosis via inflammatory and systemic mechanisms^6,18,35^. Clinical studies have also documented Gal-3 as a biomarker of cardiac dysfunction via inflammatory mechanisms^36^. In this study, we investigated the relationship between AKI and inflammation. We were aware that the uremic effect of acute renal failure may mask the systemic mechanism of AKI. We have also previously reported the deleterious role of Gal-3 in a model of unilateral ureteral obstruction, which led to renal damage independently of renal dysfunction^6^. Therefore, a systemic response may be triggered by renal injury.

Tumor models have demonstrated that cell-to-cell interactions across different localizations are mediated by vascular mechanisms and endothelial-specific pathways^37^. Renal endothelial pathophysiology is complex and involves multiple pathways^38^. Among them, the activation of systemic immune cell infiltration follows endothelial activation^11^. Recent studies demonstrated a direct interaction between the endothelial dysfunction marker CD146 and Gal-3, which induces the secretion of a distinct cytokine profile by endothelial cells, that promotes metastasis progression^39^. Our study demonstrates for the first time an in vivo interaction between these two proteins in the kidney after AKI. The consequences of an increased CD146 expression in the activated endothelium are well documented in the literature. Indeed, increased endothelial CD146 expression was associated with endocapillary proliferation, proteinuria, and inflammation in biopsies from patients suffering from different types of nephropathies^40^. Furthermore, increased CD146 expression was consistent with changes in renal morphometry during the progression of chronic renal failure. Notably, the role of CD146 in renal pathophysiology has been studied in experimental inflammatory nephropathy. Indeed, we have previously reported that endothelial-specific deletion of CD146 reduced glomerular damage in experimental glomerulonephritis by limiting inflammatory infiltrates within glomerular and interstitial activated endothelium^41^. In accordance, previous studies have reported that endothelial CD146 is involved in the regulation of monocyte trans endothelial migration in vitro and in vivo^42,43^. Furthermore, increased expression of the soluble CD146 isoform has been associated with acute kidney transplant rejection, highlighting the need to investigate the role of CD146 in AKI and its potential links to systemic response.

The interaction between Gal-3 and CD146 is not the only player in the inflammatory response. Since extracellular Gal-3 interacts with several cell surface receptors, multiple other systemic and non-systemic pathways are involved^2,3^. This has increased the interest in developing therapeutics against this lectin. Some experimental models in rodents have demonstrated the reduction of renal injury by Gal-3 inhibitors^8^. However, clinical evidence of improved renal outcomes is still unclear. Current Gal-3 inhibitors, such as MCP, lack specificity, and some side effects have been reported^44^. Consequently, to reduce the severity of inflammatory responses and renal damage caused by acute renal injury, an alternative approach could involve targeting the Gal-3/CD146 interaction to enhance the specificity of systemic pathway inhibition involving Gal-3. In conclusion, our study highlights the pivotal role of Gal-3 in AKI by initiating a systemic inflammatory response, potentially triggering a systemic cascade that promotes CD146-mediated endothelial interaction, cytokine secretion, and immune cell infiltration in renal tissue, further contributing to organ fibrosis and subclinical dysfunction (Figure S10). However, additional experiments are necessary to assess the precise role of Gal-3 in renal injury via systemic versus local effects or dependent versus independent of its binding with CD146. To this end, the generation of transgenic animals with Gal-3 specific deletion in distinct renal cell types will be of high interest. Although further work is necessary to investigate the molecular mechanisms of this interaction, our study suggests that the development of therapeutic devices against Gal-3 could be of interest to improve renal damage and systemic consequences after AKI.

## Materials and methods

All the methods were carried out in accordance with relevant guidelines and regulations. Detailed methods of all the procedures can be found in the Supplementary Information Files.

### Animals

Two- to 4-month-old male C57BL/6J Wilde-Type (WT) and Gal3-3 knock-out mice (Gal-3-/-) (Janvier laboratory, Le Genest-Saint-Isle, France) were used^6,17^. Control animals were WT littermates of knockout mice. Renal ischemia-reperfusion injury was performed as previously described^6^. In separate experiments, mice were injected with recombinant Gal-3. Mice were euthanized by pentothal overdose after 24, 48 h, and 28 days of reperfusion, and tissue and blood samples were collected for various analyses.

For bone marrow transplantation, 2-month-old male mice were irradiated at 10 grays (2 × 5 grays at 5-h intervals) with a filter, using a Faxitron irradiator (Faxitron, Tuscon, Arizona). After the second irradiation, two pairs of mice were transplanted with 10 to 15 million BM cells from a WT or a Gal-3-/- mouse. At the end of the protocol, 2 groups of chimeric mice were obtained: WT mice grafted with Gal-3-/- BM (WT^Gal−3−/− BM^) and Gal-3-/- mice grafted with WT BM (Gal-3-/-^WTBM^)^6^. These chimeric mice underwent right nephrectomy and left rIR injury, as described previously 3 months after irradiation. Sham mice underwent the same procedure, except for right nephrectomy and left rIR, and were euthanized after 28 days. In the rIR groups, mice were sacrificed at two points: 48 h and 28 days after rIR, with 6 to 9 mice per group analyzed at each time point (Table S1). All mouse parameters are detailed in Table S1.

All animal procedures were performed following the relevant guidelines and regulations approved by the French National Institute for Health and Medical Research (INSERM) and the University of Paris.

### Renal morphometry and immunostainings

Kidneys were fixed in 4% paraformaldehyde, followed by embedding in paraffin and sectioning into 6-µm slices. For frozen renal sections, tissue slices were first fixed in 4% paraformaldehyde and then embedded in Tissue-Tek O.C.T. (Sakura Finetek, Villeneuve d’Ascq, France), before being frozen in liquid isopentane.

Tubular damage was evaluated semi-quantitatively in blind by 2 different investigators using Periodic Shiff Acid (PAS)-stained slides as previously reported^6^. Briefly, we used the following scale: 0, no tubular damage; 1, damage in 1–25% of the tubules analyzed; 2, damage in 26–50% of the tubules analyzed; 3, damage in 51–75% of the tubules analyzed; 4, damage in > 76% of the tubules analyzed, and the mean value was calculated for each mouse. Tubular injury was evaluated as simultaneous quantification of both tubular dilation and necrosis in the cortex and the cortico-medullar junction of the kidney. All images were acquired on a Leica microscope (Leica Microsystems, Rueil Malmaison, France) and recorded for further analysis.

Immunostainings were performed on cryosections fixed with 4% PFA or on paraffin-embedded kidneys. Images were analyzed using a Zeiss confocal microscope. Specific primary and secondary antibodies, along with their respective dilutions, are mentioned in Table S2.

### Protein analysis

For Western blot analysis, tissues were homogenized in cell lysis buffer (50mM Tris HCl pH7.4, 1mM EDTA, 150 mM NaCl). After centrifugation, soluble proteins were quantified with the Pierce BCA Protein Assay Kit (Thermo Fischer Scientific, Courtaboeuf, France). Proteins (20 µg) were separated on 4%-20% SDS-PAGE gels and transferred onto nitrocellulose membranes (Protan, Paris, France). Blots were probed overnight at 4 °C with antibodies directed against the protein of interest. All blots were normalized with GAPDH (1/5000; Millipore, Molsheim, France). Blots were incubated with secondary horseradish peroxidase-conjugated antibodies for 1 h at room temperature. All primary and secondary antibodies used are detailed in Table S2. Chemiluminescent signals (ECL Prime, GE Healthcare) were recorded using a ChemiDoc (BioRad) and quantified with FIJI software^6^. Quantification was performed using densitometry analysis using ImageJ software with the subtraction of background intensity for each lane. The normalization was performed by dividing the intensity of the target protein band by the intensity of the corresponding loading control band for each sample. This accounts for any variations in loading or transfer efficiency.

### Plasma assays

To evaluate the balance between pro- and anti-inflammatory mediators, three pro-inflammatory cytokines (Interleukin (IL)-1β, Tumor Necrosis Factor (TNF)-α and IL-6), as well as IL-10, were measured in the plasma of mice using ELISA kits (Invitrogen^®^ #88–7013 A-76; #88–7064 A-22; #88-7105-22; #88-7324-22). Plasma and urine Gal-3 levels were measured using an ABCAM^®^ ELISA (ab203369). Blood urea nitrogen (BUN) and plasma creatinine levels were measured with an enzymatic method (Konelab automater) and expressed in mM and µM, respectively.

### Statistical analysis

We hypothesized that ischemic AKI would induce an increase in Gal-3 protein expression from 0.1 to 0.3 (SD 0.1) based on our previously published studies^6,18^. Using an α risk of 5% and a power of 90% at least 3 animals per group were required. However, more mice were added in groups to strengthen the result and to compensate the heterogeneity of our rIR experimental model. Data are expressed as mean ± SEM. Comparisons between groups were performed using two way-ANOVA with Bonferroni correction. All analyses were performed using GraphPad Prism V9 (GraphPad Software, USA), with statistical significance defined as *P* < 0.05 for all between and within group differences.

## Supplementary Information

Below is the link to the electronic supplementary material.

Supplementary Material 1

## Data Availability

All data generated or analyzed for this study are included in this article and its Supplementary Information files. All materials and protocols are available from the corresponding author upon reasonable request.
