# Supplementary material for: Galectin-3/CD146 interaction promotes renal damage and systemic inflammation after acute kidney injury
Source: Sci Rep. 2025 Nov 24;15:41575. doi: 10.1038/s41598-025-25477-4 (PMC12644692; doi:10.1038/s41598-025-25477-4)
Supplement: Supplementary file 1 — Supplementary Material 1 [file 41598_2025_25477_MOESM1_ESM.pdf]

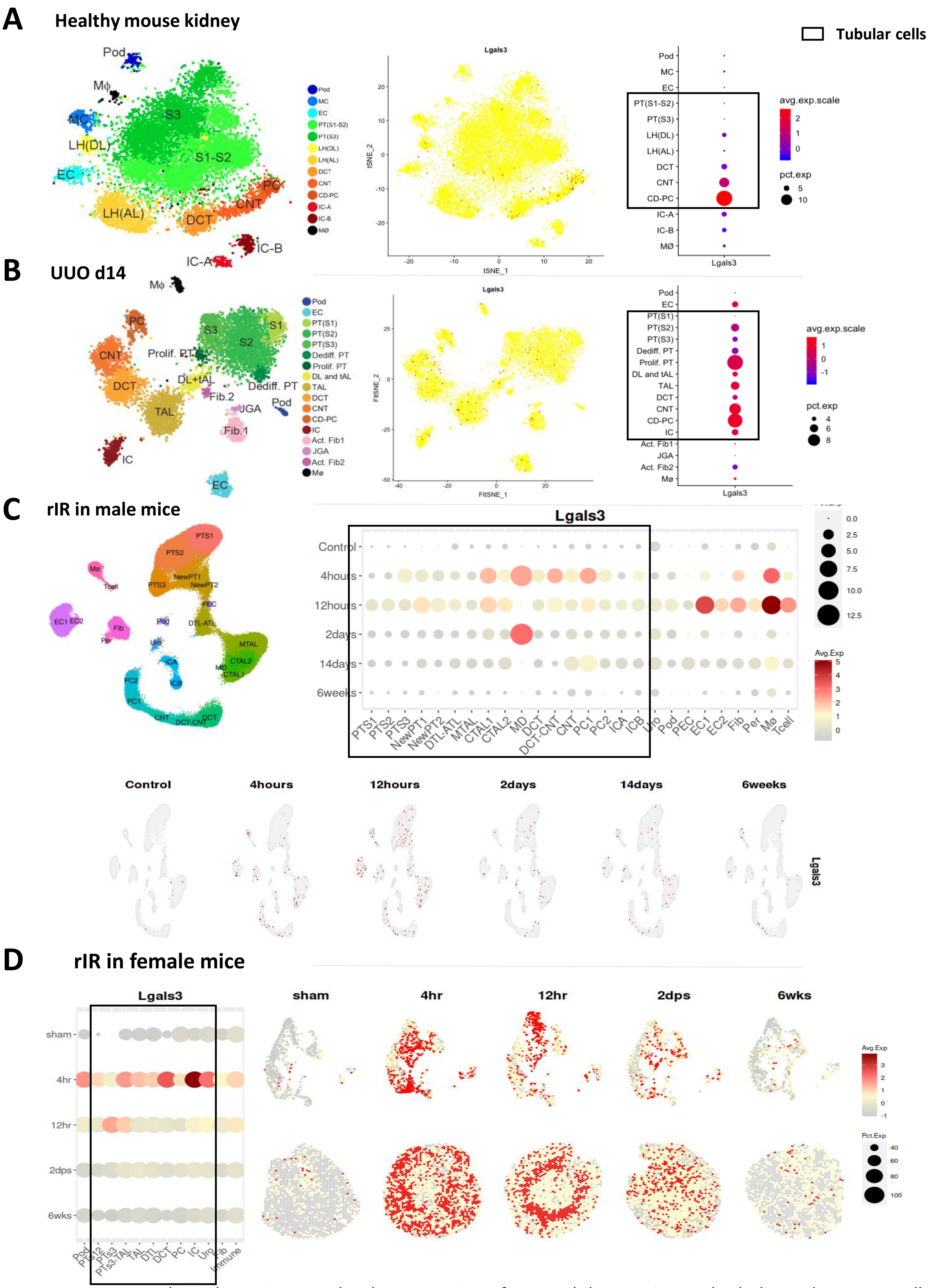

Figure S1: scRNAseq analysis shows increased Gal-3 expression after renal damage in renal tubular and immune cells. The expression profile of Gal-3 was assessed using Humphrey's lab scRNAseq dataset and software in healthy mouse kidney (A), after 14 days of UUO (B), after rIR from 4 hours to 6 weeks in male (C), and female mice (D). Copyright (c) 2021 Washington University in St. Louis, MO. Results were obtained from http://humphreyslab.com/SingleCell/ (H. Wu, et al, J Am Soc Neph, 2018; 29(8):2069-2080))

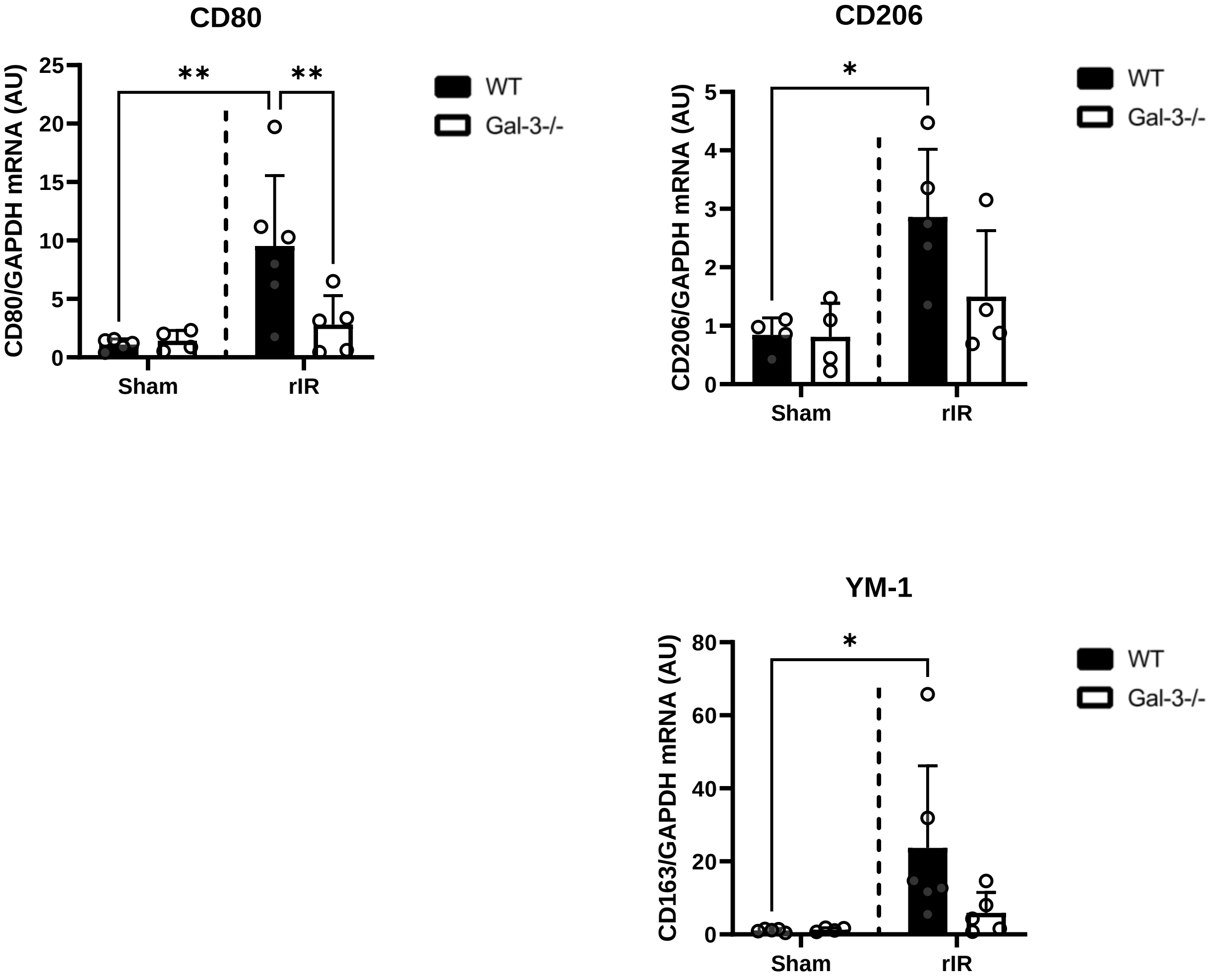

Figure S2: mRNA expression of macrophage polarization markers CD80 (M1), CD206 and CD163 (M2) was measured by qPCR, in WT and Gal-3-/- mice after 48 hours of renal IR. Data are presented as mean ± SEM (n=4-6). Two-way ANOVA corrected with Bonferroni transformation; *P<0.05, **P<0.01, and ***P<0.001.

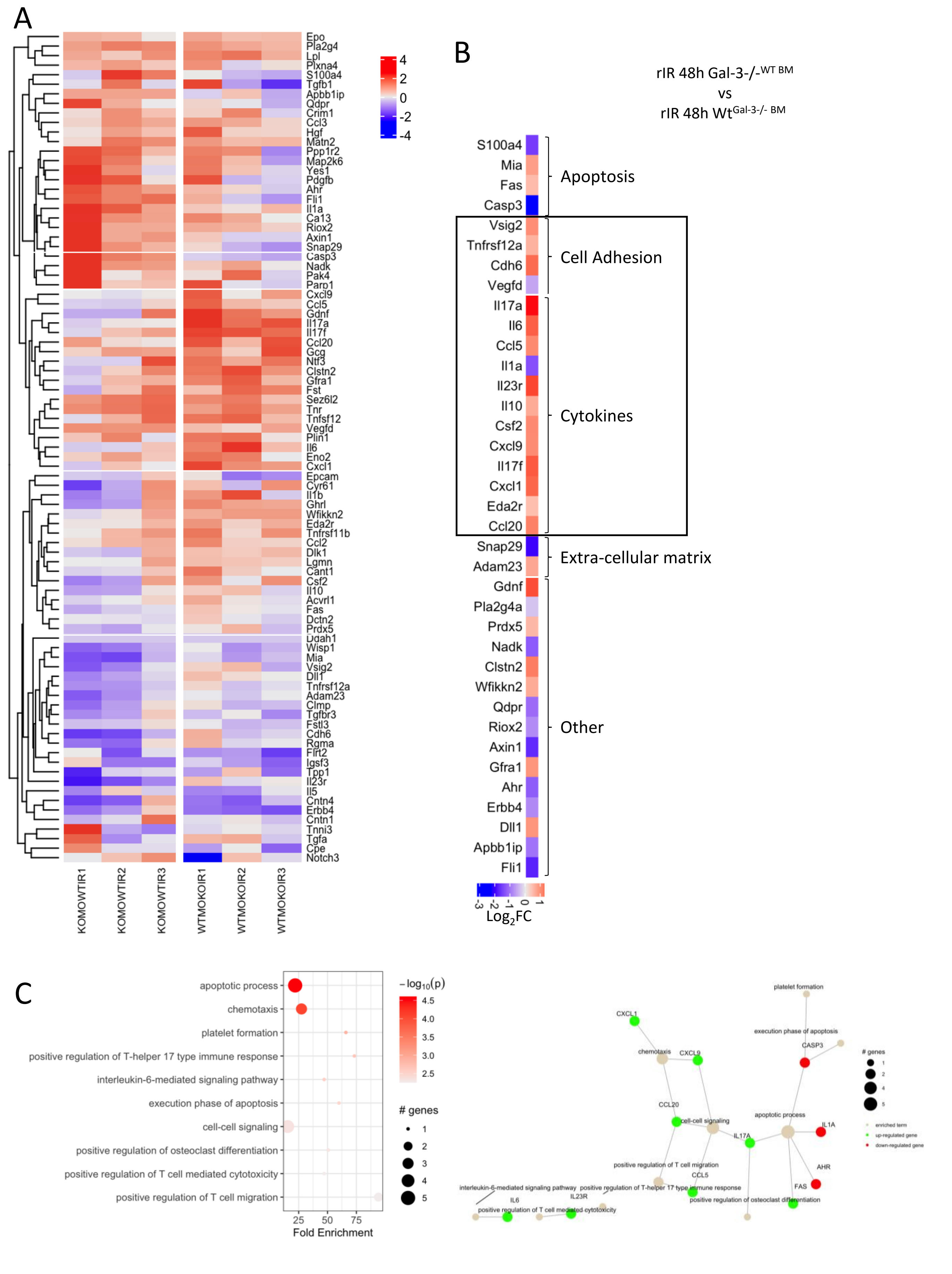

Figure S3: Relative quantitative panel of multiple plasma protein expression according to genotype and rIR in WT$^{Gal\text{-}3\text{-}/\text{-}BM}$ and in Gal-3-/-^{WTBM} mice after 48h of rIR (A). Relative quantitative panel of significantly differentially expressed proteins between WT$^{Gal\text{-}3\text{-}/\text{-}BM}$ and Gal-3-/-^{WTBM} mice after 48h of rIR (B). Enrichment analysis and map based on ontology library for WT$^{Gal\text{-}3\text{-}/\text{-}BM}$ 48h vs Gal-3-/-^{WTBM} mice (C) (n=3-4 mice per group).

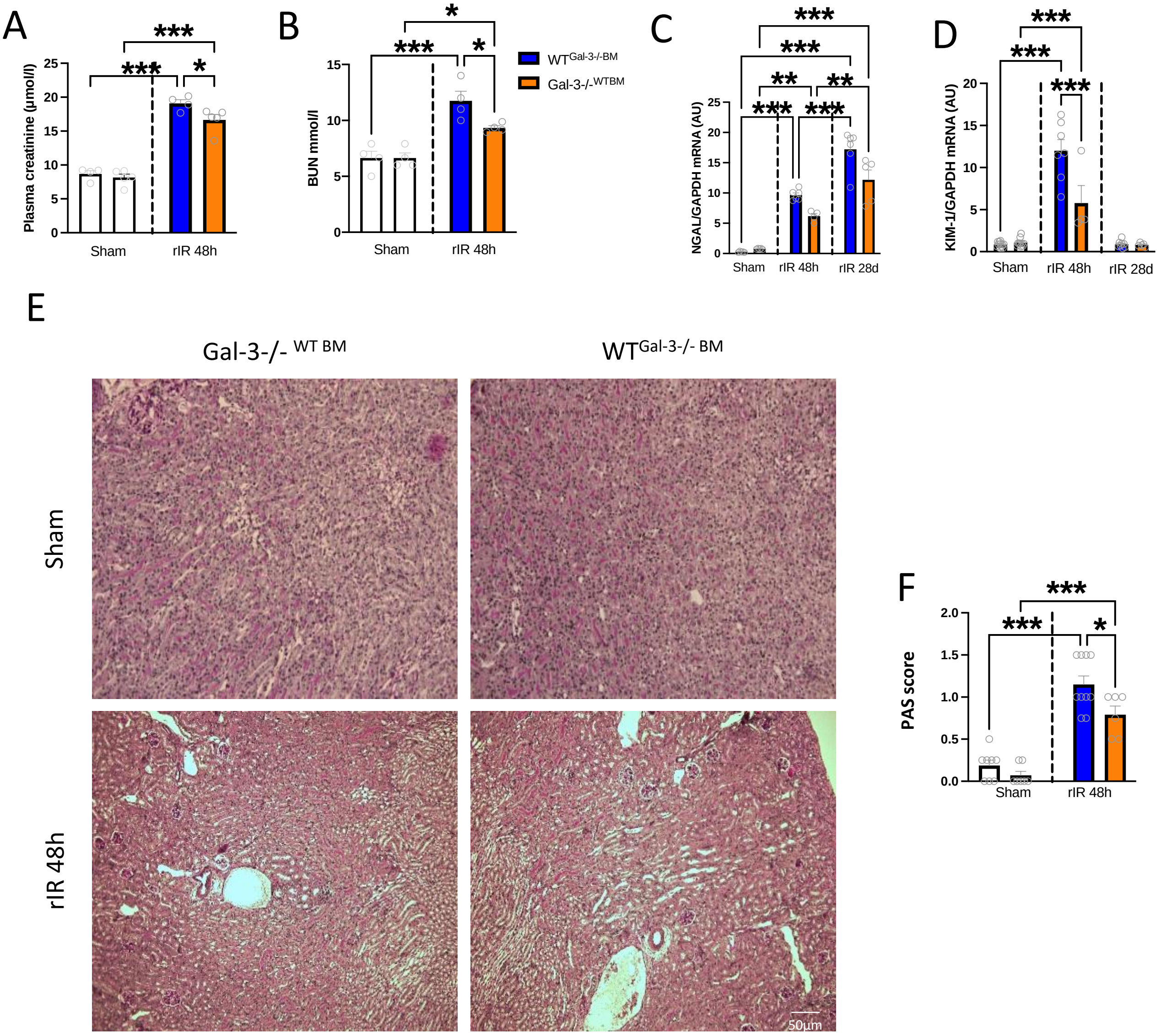

Figure S4: Irradiated mice of both genotypes underwent adoptive transfer to assess whether Gal-3 originated from renal tissue or immune cells promoted damage after rIR. WT$^{Gal\text{-}3\text{-}/\text{-}BM}$ (Gal-3 only in renal tissue) and Gal-3-/-^{WTBM} (Gal-3 only in bone marrow-derived cells). Plasma creatinine levels (A) and BUN (B) after 48h of rIR. mRNA expression of kidney injury markers NGAL (C) and KIM-1 (D) within damaged kidneys after 48h and 28d of rIR. The histological evaluation included renal PAS staining (E) and scoring for kidney damage (F) at 48h post-rIR. Data are presented as mean ± SEM (n=4-12). Two-way ANOVA corrected with Bonferroni transformation; *P<0.05, **P<0.01, ***P<0.001. Scale bar, 50 µm.

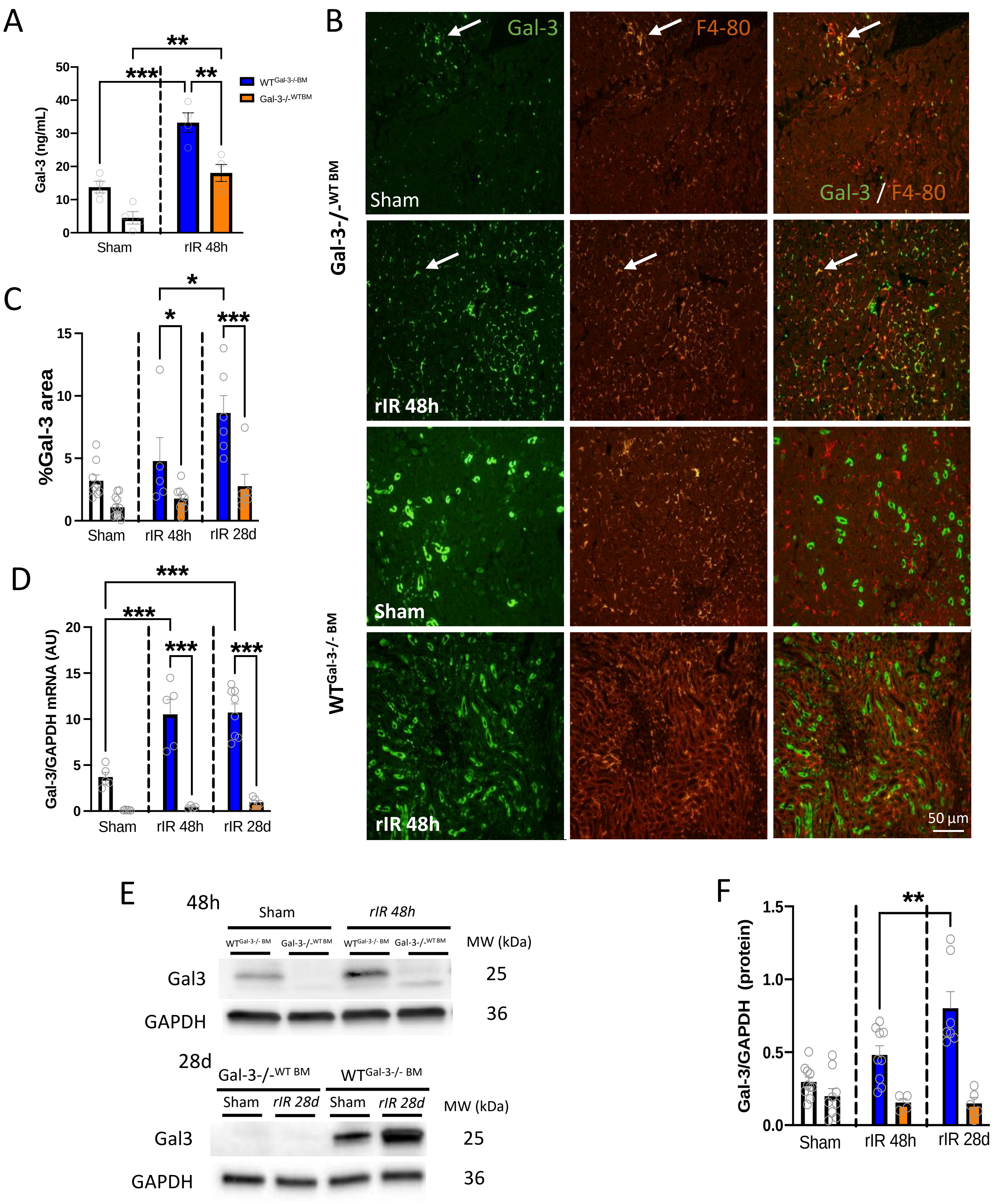

Figure S5: Gal-3 plasma levels at 48 hours post-rIR (A). Co-immunostaining between Gal-3 (in green) and F4-80 (in red) in renal tissue after 48h of rIR (B). Quantification of kidney Gal-3 histological expression at 48h and 28d post-rIR (C). Renal Gal-3 mRNA expression (D) and Western blot analysis of Gal-3 (E), with quantification (F) in kidneys at 48h and 28d post-rIR. Data are presented as mean ± SEM (n=4-12). Two-way ANOVA corrected with Bonferroni transformation; *P<0.05, **P<0.01, ***P<0.001. Scale bar, 50 µm.

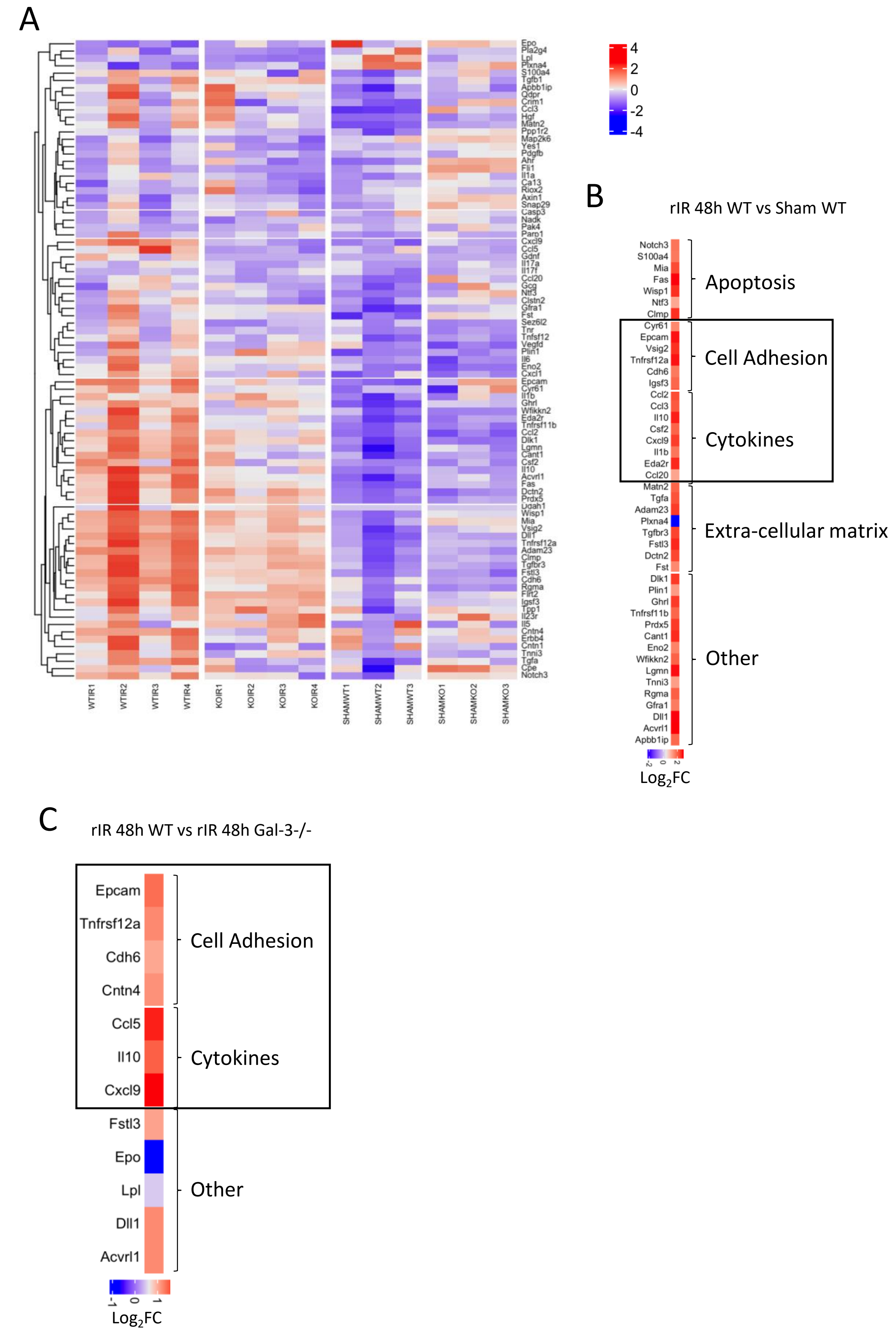

Figure S6: Relative quantitative panel of multiple plasma protein expression according to genotype after rIR for mice (A). A relative quantitative panel of significantly differentially expressed protein between rIR 48h WT mice and Sham WT mice (B), and WT mice and Gal-3$^{-/-}$ mice after 48h of IR (C) (n=3-4 mice per group).

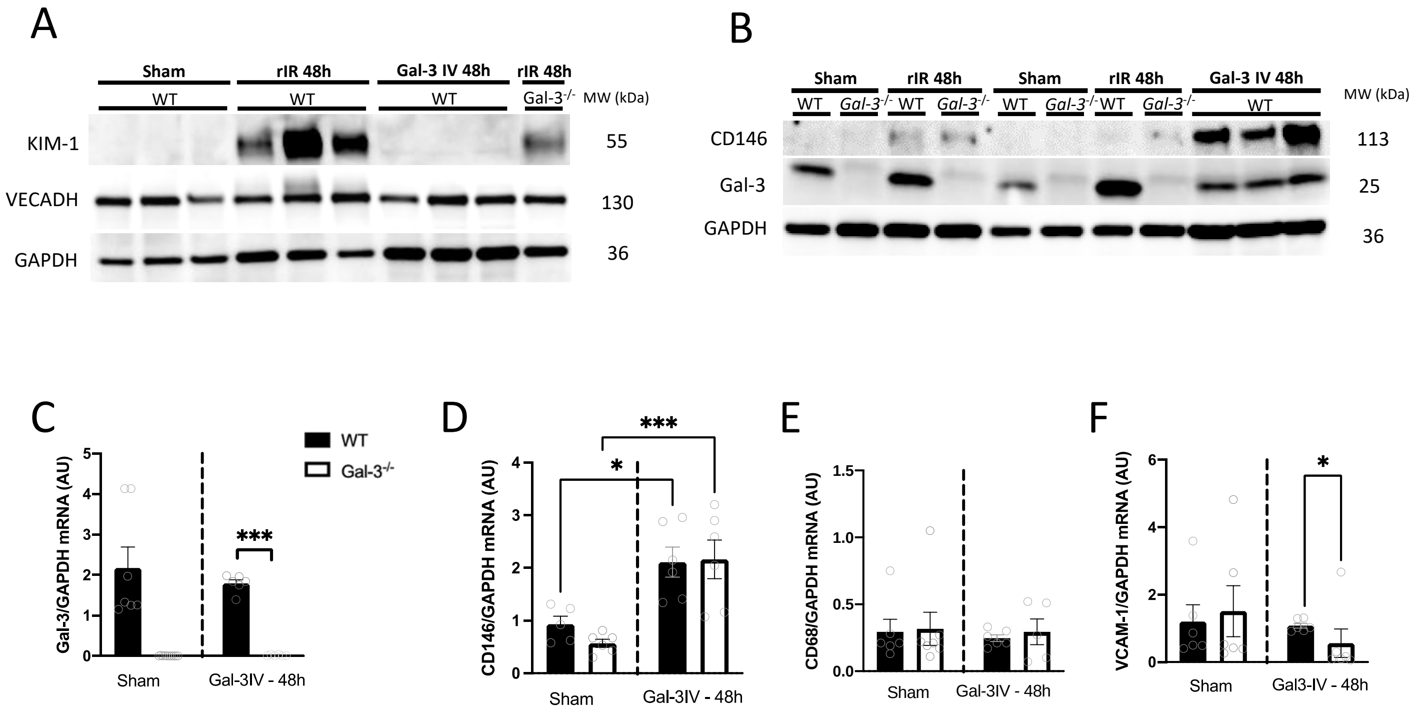

Figure S7: Kidney protein expression of KIM-1 and VEcadherin (VECADH) (A), CD146 and Gal3 (B), after 48h of rIR or Gal-3 recombinant injection in WT and Gal-3-/- mice. mRNA expression of Gal-3 (C), CD146 (D), CD68 (E), and VCAM-1 (F) were measured by qPCR, after Gal-3 injection in WT and Gal-3-/- mice. Data are presented as mean ± SEM (n=5-7). Two-way ANOVA corrected with Bonferroni transformation; *P<0.05, **P<0.01, and ***P<0.001.

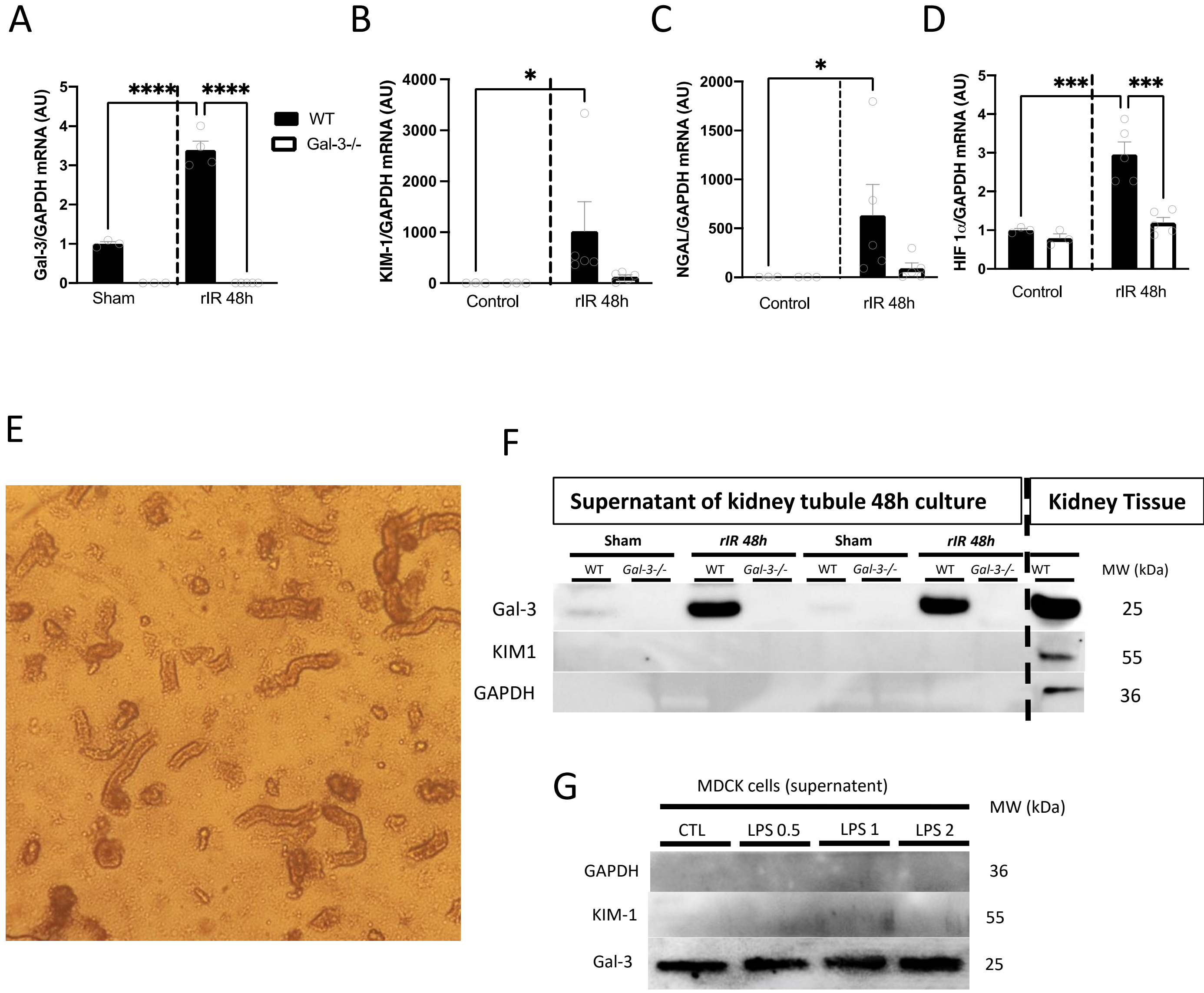

Figure S8: mRNA expression of Gal-3 (A), KIM-1 (B), NGAL (C), and HIF1-α (D), in isolated tubules from WT and Gal-3-/- mice, after 48h of rIR. Representative image of renal tubules after extraction (E). Protein expression of Gal-3, KIM-1, and CD146 in tubular culture supernatants (F) and tissue from kidneys, 48 post-rIR of WT and Gal-3-/- mice. Protein expression of Gal3, KIM-1, and GAPDH in supernatants of cultured tubular cell lines after LPS stimulation (G). Data are presented as mean ± SEM (n=3-6). Two-way ANOVA corrected with Bonferroni transformation; *P<0.05, **P<0.01 and ***P<0.001.

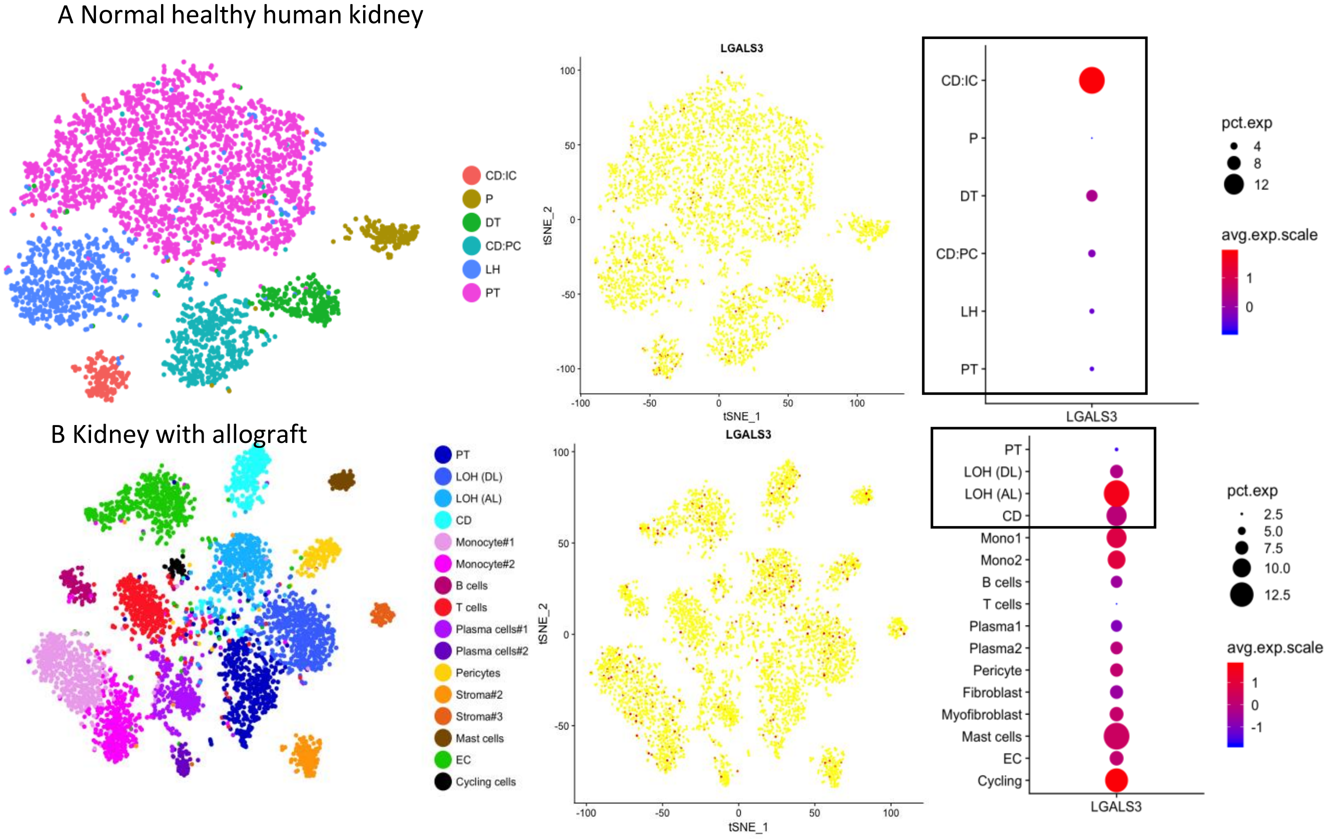

Figure S9: Kidney scRNAseq analysis of Gal-3 using Humphrey's Lab data in normal healthy human kidney (A), and human kidney with allograft (B). Copyright (c) 2021 Washington University in St. Louis, MO. Results obtained from http://humphreyslab.com/SingleCell/ (H. Wu, A.F. Malone, E. Donnelly, Y. Kirita, K. Uchimura, S.M. Ramakrishnan, J. Gaut and B.D. Humphreys. Single-cell transcriptomics of a human kidney allograft biopsy defines a diverse inflammatory response. J Am Soc Neph, 2018; 29(8):2069-2080)).

Gal-3 promotes systemic inflammatory response through interaction with CD146, modulating cytokine secretion

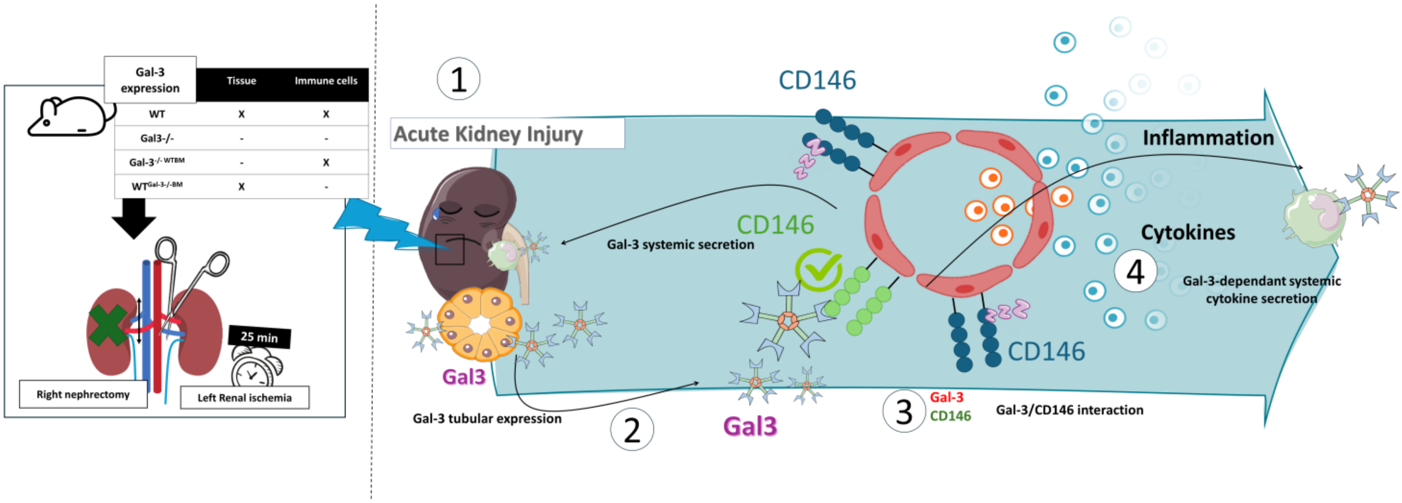

Figure S10: A schematic representation suggesting the deleterious role of Gal-3 in the kidney and further systemic inflammation after acute kidney injury.

Supplementary methods

Renal ischemia-reperfusion injury

Briefly, a right nephrectomy and left renal pedicle occlusion (25 min of ischemia), followed by reperfusion, were performed under anesthesia (intraperitoneal injection of ketamine: 100 mg/kg and xylazine: 20 mg/kg). Sham mice underwent the same procedure, except for renal pedicle occlusion and right nephrectomy. To understand the kinetics after rIR, mice were euthanized at 24h, 48h, and 28d after reperfusion. We analyzed 6 to 12 mice per group, and their details can be found in **Table S1**.

Galectin-3 treatment

For Gal-3 treatment, 0.2 mg/kg of recombinant mouse Gal-3 (9039-GAB; R&D system, USA) diluted in 200 µL of PBS 1X, was injected intravenously (retro-orbital) in mice under isoflurane inhalation (2%). This dose was justified according to previous publications showing systemic effect 48 hours after 0.2 mg/kg intraperitoneal injection[19]. Sham mice were littermates intravenously injected with vehicle of 200 µL PBS 1X. Mice were sacrificed 48h and 28d after Gal-3 injection. Six to 12 mice per group were used for this study **(Table S1)**.

Tubular extraction

Renal tissue was incubated in lysis buffer (Collagenase Type I and Roswell Park Memorial Institute medium glutamine free) for 25 minutes at 37°C. Tissue was dissociated using MACs filter (70µm and 100µm). Then, tubules were cultured for 24 or 48 hours in a Krebs-Henseleit solution.

Cell culture

MDCK Cells:

The cells were cultured for one week in a 6-well plate until confluence, in DMEM - Ham's F12 (1:1) with 10% FBS. They were stimulated for 24 hours at different doses of LPS in serum-free medium: 0.5 µg/µL, 1.0 µg/µL, and 2.0 µg/µL. The supernatant was then collected and concentrated using centrifuge filters (Microcon YM-3, 42403). Two rounds of centrifugation were performed at 14,000g for 12 minutes, followed by one round at 1,000g for 3 minutes to recover the concentrated proteins.

mPTEC Proximal Tubular Cells:

The kidneys from WT mice were collected and the cortex was separated from the medulla. After digesting the cortex with collagenase, the solution was first passed through a 250 µm filter and then through an 80 µm filter. mPTEC cells were isolated and cultured in a 12-well plate for 7 days in a medium with 5% serum. On the 7th day, the cells were stimulated with TGF-β (Human TGFβ1, Miltenyi Biotech) at 5 ng/mL in a medium with 1% FBS. The supernatants were collected 24 hours later and concentrated simultaneously with the supernatants from the MDCK cells.

Gene expression analysis

For RT-qPCR analyses, total RNA extraction from tissue, reverse transcription and qPCR were performed as previously described[6]. mRNA levels for genes of interest were normalized to the glyceraldehyde-3-phosphate dehydrogenase (GAPDH) mRNA levels and expressed as the relative change compared to the control samples. All primers used for the study are listed in **Table S3**.

Proteomic analysis

Mice plasma samples were sent to Olink Proteomics (Watertown, MA) for analysis. They were processed using Olink's Proximity Extension Assay (PEA) technology, which employs antibody pairs linked to unique oligonucleotides and quantified through PCR. The selected panel was Olink Mouse exploratory (v.3801). The oligonucleotides are brought into proximity when both antibodies bind the target protein simultaneously, allowing for hybridization. Afterward, the oligonucleotide sequence is

extended by DNA polymerase, amplified, and measured by qPCR to assess the initial protein levels in the samples. Raw analyte expression values obtained through PCR underwent multiple transformations by Olink, including a log2 transformation, resulting in normalized protein expression (NPX) values. NPX values are not absolute quantifications, but an indication of the relative concentration of each analyte. The difference in the proteins between case-control comparisons was evaluated using the Mann-Whitney test. P-values were adjusted with the Benjamini-Hochberg method. Differences were expressed using Log2 fold change. Additionally, protein interactions were analysed using enrichment analysis with Gene Ontology library.

Co-immunoprecipitation (CO-IP)

For CO-IP, proteins were extracted using a low detergent buffer (150mM NaCl, 50mM TriHCl, 1% Triton X100, pH = 8). Recombinant protein or lysate protein was incubated with a specific antibody (detailed in **Table S2**) and a cocktail of Protein G and Protein A for 24h at 4°C. The mixture was applied in a column with magnetic beads and a magnetic field. Columns were washed 4 times with wash buffer (20mM TrisHCl, pH = 7.5). Elution was performed using LDS sample buffer (NuPAGE®) preheated at 95°C. Proteins were identified using Western Blot as previously described.

scRNAseq analysis

For scRNAseq (Single Cell RNA sequencing) analysis, all data were obtained and analyzed from Humphrey's lab software (https://humphreyslab.com), Copyright (c) 2021 Washington University in St. Louis, MO. The analysis was performed in accordance with the terms of use of Humphrey's lab software, using the LGLS3 reference. Datasets were kidney healthy mice and kidney after ureteral occlusion, kidney mice after rIR, female kidney mice after renal ischemia-reperfusion for spatial transcriptomics, human normal adult kidney, and after allograft.

Table S1: Mice characteristics. Value expressed in median [Q1, Q3]

	Sham WT			Sham Gal-3-/-			rIR WT			rIR Gal-3-/-		
Time-point	24h (n=7)	48h (n=6)	28d (n=9)	24h (n=6)	48h (n=9)	28d (n=8)	24h (n=8)	48h (n=12)	28d (n=10)	24h (n=8)	48h (n=6)	28d (n=12)
Weight before surgery	28.6 [27.6, 29.4]	21.2 [20.6, 25.4]	27.4 [26.8, 28.7]	28.0 [27.8, 28.5]	25.6 [24.7, 27.9]	26.3 [24.2, 27.8]	29.4 [27.2, 30.1]	25.8 [25.4, 27.3]	25.0 [23.5, 26.0]	29.4 [28.3, 30.8]	27.0 [25.6, 28.4]	26.3 [24.2, 27.8]
Weight after surgery (mg)	27.2 [26.5, 28.5]	20.7 [20.5, 24.3]	28.0 [27.1, 28.1]	26.7 [25.9, 27.5]	26.1 [24.4, 28.0]	26.4 [22.6, 28.0]	26.4 [26.0, 28.2]	24.6 [23.5, 27.4]	26.0 [24.4, 27.6]	26.4 [25.1, 27.9]	27.4 [25.4, 28.0]	26.4 [22.6, 28.0]
Heart weight / femoral length (mg/mm)	7.4 [6.9, 9.5]	6.9 [6.4, 7.4]	7.9 [7.5, 8.3]	8.8 [8.5, 9.0]	6.7 [6.2, 9.3]	7.1 [5.6, 7.6]	7.4 [7.0, 8.7]	7.4 [7.1, 9.7]	7.1 [7.0, 7.5]	8.5 [7.4, 8.6]	7.4 [6.8, 8.0]	7.1 [5.6, 7.6]
Kidney weight / femoral length (mg/mm)	8.5 [7.5, 9.6]	7.0 [6.7, 8.6]	7.7 [7.4, 8.1]	9.3 [8.5, 10.2]	7.2 [6.9, 9.0]	8.2 [6.6, 8.6]	11.6 [9.3, 13.5]	10.3 [8.0, 11.1]	7.8 [7.6, 8.2]	8.9 [8.3, 9.5]	8.8 [8.0, 9.5]	8.2 [6.6, 8.6]
Spleen weight / femoral length (mg/mm)	3.8 [3.1, 5.2]	4.6 [4.4, 5.0]	4.7 [3.9, 5.5]	5.6 [4.8, 6.5]	3.0 [3.0, 4.3]	4.9 [4.6, 5.3]	3.1 [2.6, 3.6]	4.1 [4.0, 4.6]	4.3 [4.2, 4.4]	3.3 [3.0, 3.5]	3.7 [3.0, 4.0]	4.9 [4.6, 5.3]
Lung weight / femoral length (mg/mm)	10.1 [9.8, 11.0]	9.3 [8.2, 10.1]	10.4 [9.2, 20.5]	10.0 [9.3, 12.0]	9.8 [7.6, 10.4]	9.0 [7.7, 13.3]	10.4 [9.3, 13.9]	10.6 [9.4, 13.4]	7.9 [7.1, 9.0]	9.8 [9.0, 14.7]	14.2 [13.3, 15.0]	9.0 [7.7, 13.3]
Liver weight / femoral length (mg/mm)	58.1 [56.3, 63.2]	63.8 [53.1, 67.0]	72.7 [67.0, 74.8]	66.6 [64.2, 70.9]	61.7 [55.8, 65.7]	61.5 [52.4, 65.3]	61.5 [54.1, 70.6]	58.2 [54.2, 74.3]	58.2 [54.1, 63.5]	60.9 [59.5, 62.6]	61.6 [58.9, 65.1]	61.5 [52.4, 65.3]

	WT mice injected with Gal-3		Gal-3-/- injected with Gal-3	
Time-point	48h (n=6)	28d (n=4)	48h (n=6)	28d (n=4)
Weight before surgery (mg)	25.0 [25.0, 25.8]	24.6 [24.2, 25.0]	24.0 [23.2, 24.8]	31.0 [28.8, 32.0]
Weight after surgery (mg/mm)	26.1 [25.3, 26.1]	25.9 [25.2, 26.5]	24.4 [24.2, 25.4]	34.2 [31.0, 36.1]
Heart weight / femoral length (mg/mm)	7.9 [7.7, 8.3]	5.5 [5.0, 6.3]	5.2 [5.2, 6.8]	8.9 [8.0, 9.2]
Kidney weight / femoral length (mg/mm)	7.3 [7.2, 7.4]	7.1 [6.7, 7.4]	6.4 [6.3, 7.0]	9.3 [8.4, 10.1]
Spleen weight / femoral length (mg/mm)	4.7 [4.4, 5.8]	3.8 [3.7, 4.1]	4.5 [4.0, 5.3]	4.1 [3.8, 4.4]
Lung weight / femoral length (mg/mm)	10.1 [9.6, 10.7]	8.1 [7.4, 9.2]	9.4 [8.2, 11.6]	10.7 [9.8, 11.7]
Liver weight / femoral length (mg/mm)	66.8 [62.6, 67.0]	50.4 [49.8, 52.3]	63.5 [58.0, 65.9]	61.3 [57.6, 68.6]

	WT $^{Gal-3-/-BM}$			Gal-3-/- $^{WT\ BM}$		
Time-point	Sham (n=6)	48h (n=9)	28d (n=9)	Sham (n=6)	48d (n=6)	28d (n=5)
Weight after surgery (mg)	25.5 [24.8, 26.9]	19.8 [18.5, 21.5]	23.8 [23.7, 24.4]	26.2 [24.7, 26.9]	21.2 [20.4, 21.8]	25.1 [24.4, 25.7]
Heart weight / femoral length (mg/mm)	4.7 [4.6, 4.9]	4.3 [4.0, 3.7]	4.4 [4.3, 4.5]	4.8 [4.5, 5.1]	4.4 [4.3, 4.7]	5 [4.6, 5.1]
Kidney weight / femoral length (mg/mm)	7.1 [7.0, 7.7]	6.6 [6.2, 7.1]	7.8 [7.5, 8]	8 [7.3, 8.3]	6.8 [6.5, 7.8]	8.6 [8.3,8.6]
Lung weight / femoral length (mg/mm)	6.7 [6.5, 6.8]	6.7 [6.1, 7.0]	7.2 [6.9, 7.5]	6.6 [7.5, 6.9]	7.5 [7, 8]	6.5 [6, 7.3]

Table S2: Primary antibodies used for Western Blot and Immunofluorescence.

Targeted protein	Manufacturer	Ref	Dilution	Incubation	Molecular Weight (WB) / Type of tissue fixation (IF) / Fluorochrome associated
**Western Blot**					
**Galectin-3**	ABCAM	ab76245	1/1000	O/N 4°C	16 kDa
**GAPDH**	Sigma	G9545	1/1000	O/N 4°C	36 kDa
**CD146**	ABCAM	ab75769	1/1000	O/N 4°C	70 kDa
**CD68**	Santa-Cruz	SC-20060	1/1000	O/N 4°C	35 kDa
**KIM-1**	R&D	AF1817	1/1000	O/N 4°C	~ 85 kDa
**VECADHERIN**	ABCAM	ab33168	1/1000	O/N 4°C	135 kDa
**CD31**	ABCAM	ab28364	1/1000	O/N 4°C	110 kDa
**VCAM-1**	ABCAM	Ab106777	1/1000	O/N 4°C	81 kDa
**Secondary Anti-Rabbit**	Amersham	NA9340	1/10000	1h RT	
**Secondary Anti-Rat**	Amersham	NA935	1/10000	1h RT	
**Secondary Anti-mouse**	Amersham	NA9310	1/10000	1h RT	
**Immunofluorescence / Immunostaining**					
**Galectin-3**	MERK	MABT51	1/200	O/N 4°C	Paraffin
**Megalin**	Home made	-	1/4000	O/N 4°C	Paraffin
**Tamm-Horsfald**	Santa Cruz	SC-19554	1/400	O/N 4°C	Paraffin
**KIM-1**	R&D	AF1817	1/200	O/N 4°C	Paraffin
**NCCT**	Millipore	ab3553	1/500	O/N 4°C	Paraffin
**AQP2**	Home made		1/100	O/N 4°C	Paraffin
**F4/80**	ABCAM	ab6640	1/200	O/N 4°C	Frozen
**CD146**	ABCAM	ab75769	1/200	O/N 4°C	Paraffin
**aSMA**	ABCAM	ab5694	1/100	O/N 4°C	Frozen
**Megalin**	Home made	-	1/4000	O/N 4°C	Paraffin
**Tamm-Horsfald**	Santa Cruz	SC-19554	1/400	O/N 4°C	Paraffin
**Secondary Anti-Rabbit**	Thermo Fisher	A21206	1/500	45 min RT	Alexa fluor 488
	Thermo Fisher	A21207	1/500	45 min RT	Alexa fluor 594
**Secondary Anti-Rat**	Thermo Fisher	A21208	1/500	45 min RT	Alexa fluor 488
	Thermo Fisher	A21209	1/500	45 min RT	Alexa fluor 594
**CO-IP**					
**Galectin-3**	MERK	MABT51	4 µg	O/N 4°C	-
**CD146**	R&D	MAB932	4µg	O/N 4°C	-
**Protein G**	µMACS™ Protein G MicroBeads	130-071-101	50 µL	O/N 4°C	-
**Protein A**	µMACS™ Protein A MicroBeads	130-071-001	50 µL	O/N 4°C	-

Table S3: Primers used for our study.

Gene	Forward primer	Reverse primer
**CD80**	TCGTCTTTCACAAGTGTCTTCAG	TTGCCAGTAGATTCGGTCTTC
**CD163**	TCTCAGTGCCTCTGCTGTCA	CGCCAGTCTCAGTTCCTTCT
**CD206**	CCACAGCATTGAGGAGTTTG	ACAGCTCATCATTTGGCTCA
**CD68**	TTCTGCTGGGAAATGCAAG	AGAGGGGCTGGTAGGTTGAT
**Coll1a1**	GCAGGTTCACCTACTCTGTCCT	CTTGCCCCATTCATTTGTCT
**Coll3a1**	TCCCCTGGAATCTGTGAATC	TGAGTCGAATTGGGGAGAAT
**Fibronectin**	ACTGACGAAGAGCCCTTAC	AGATAACCGCTCCCATTCC
**GAPDH**	AACTTTGGCATTGTGGAAGG	ACACATTGGGGGTAGGAACA
**Gal-3**	GTGAAACCCAACGCAAACAG	CCAGTTATTGTCCTGCTTCGT
**MCP-1**	AGGTCCCTGTCATGTTCTG	TCTGGACCCATTCCTTCTTG
**NGAL**	CTGAATGGGTGGTGAGTGTGG	CTTGGTATGGTGGCTGGTGG
**KIM-1**	TCCACTCCTGTCTTTATGCTCC	GTCCCAACCTCTATCACACCTG
**CD146**	CTGCGAGGCAGAAAGTAACC	ACCCACACCTTCCTCTCCTT
**ICAM-1**	CATTTACCCTCAGCCACTTCCT	GTTCACAGTCTTGCTCCATCCA
**VCAM-1**	GCAGTCCTGTGAACCTGACC	CTAATTCCAGCCTCGTTAATCCCT

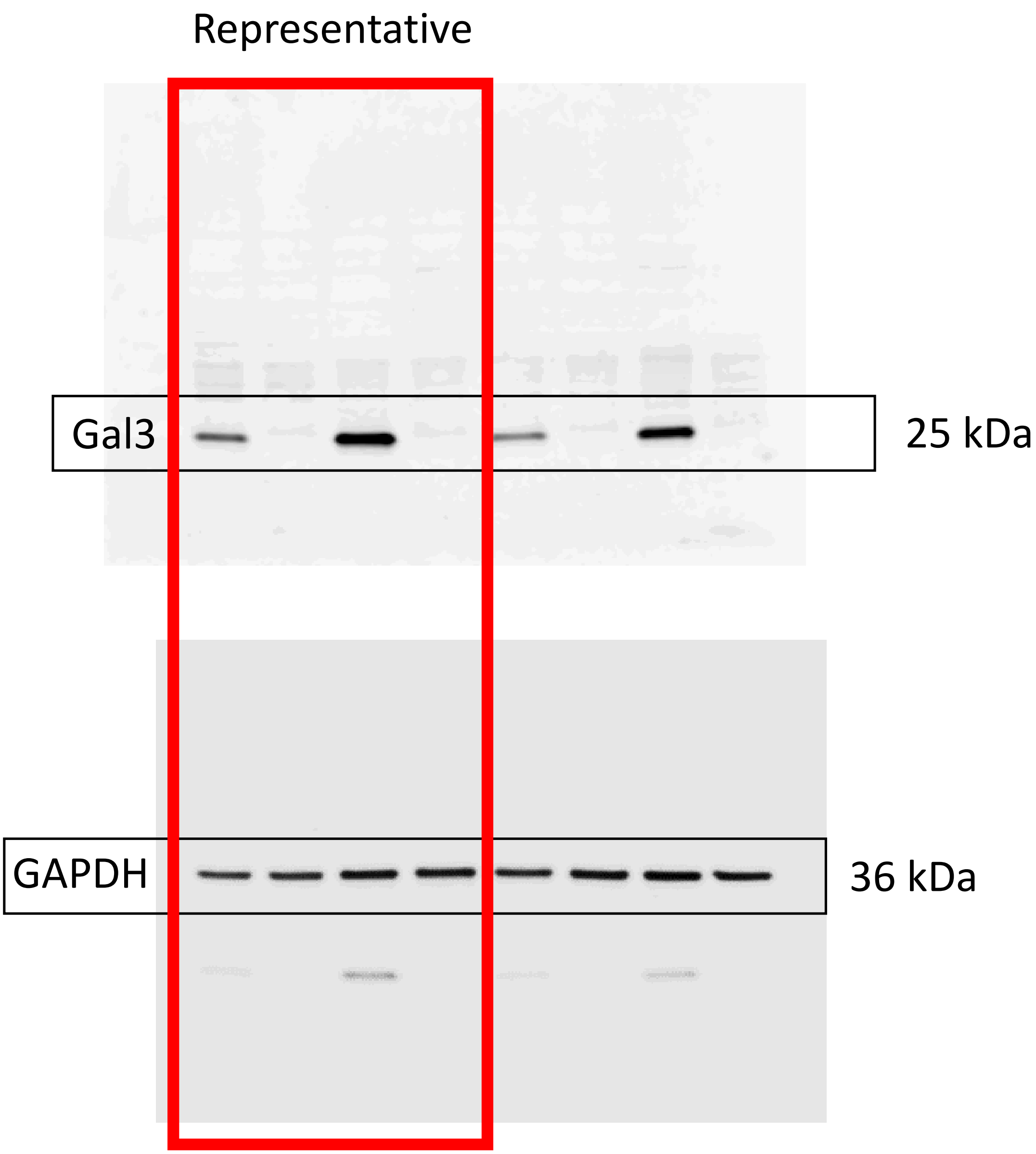

Figure 1C

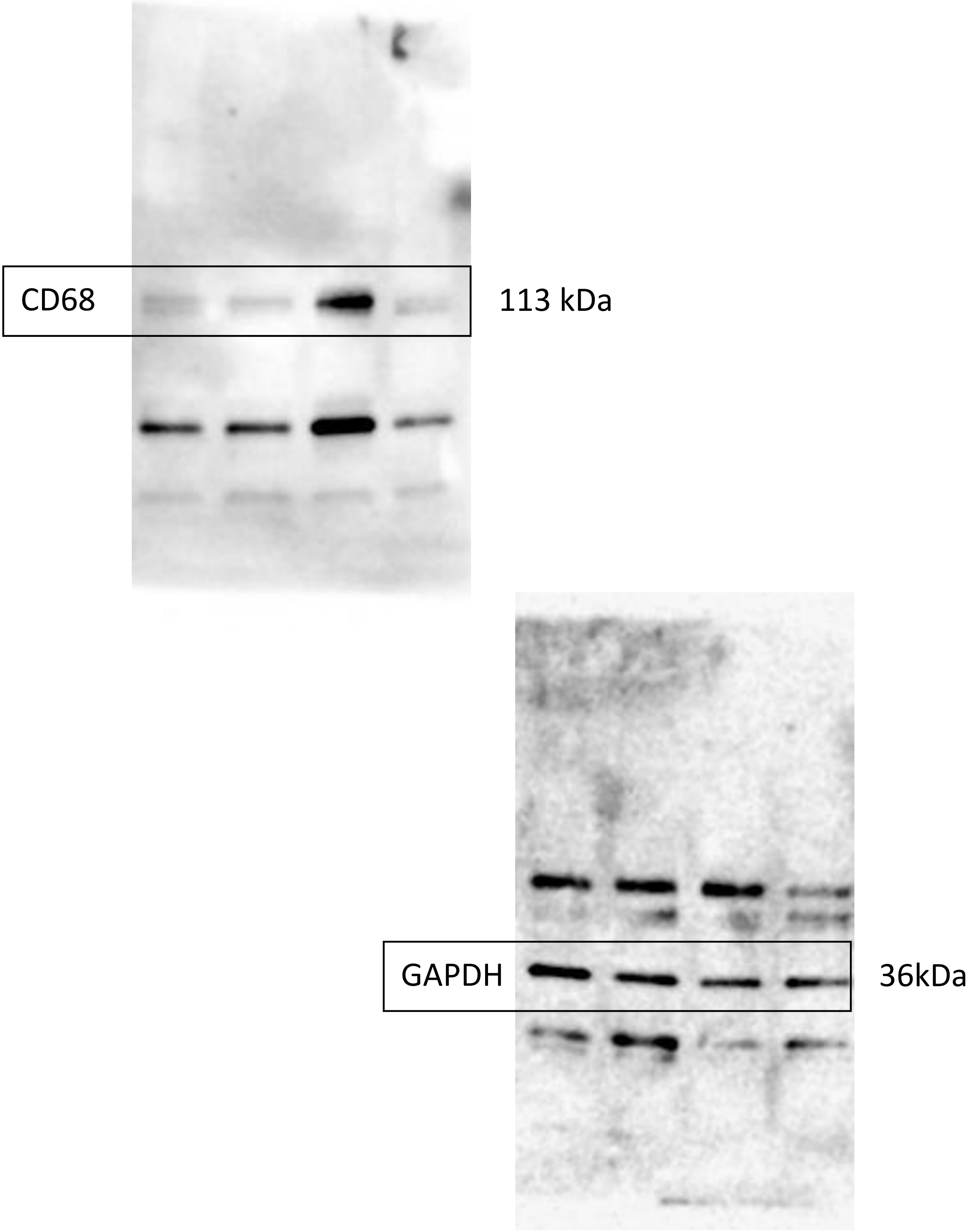

Figure 3C

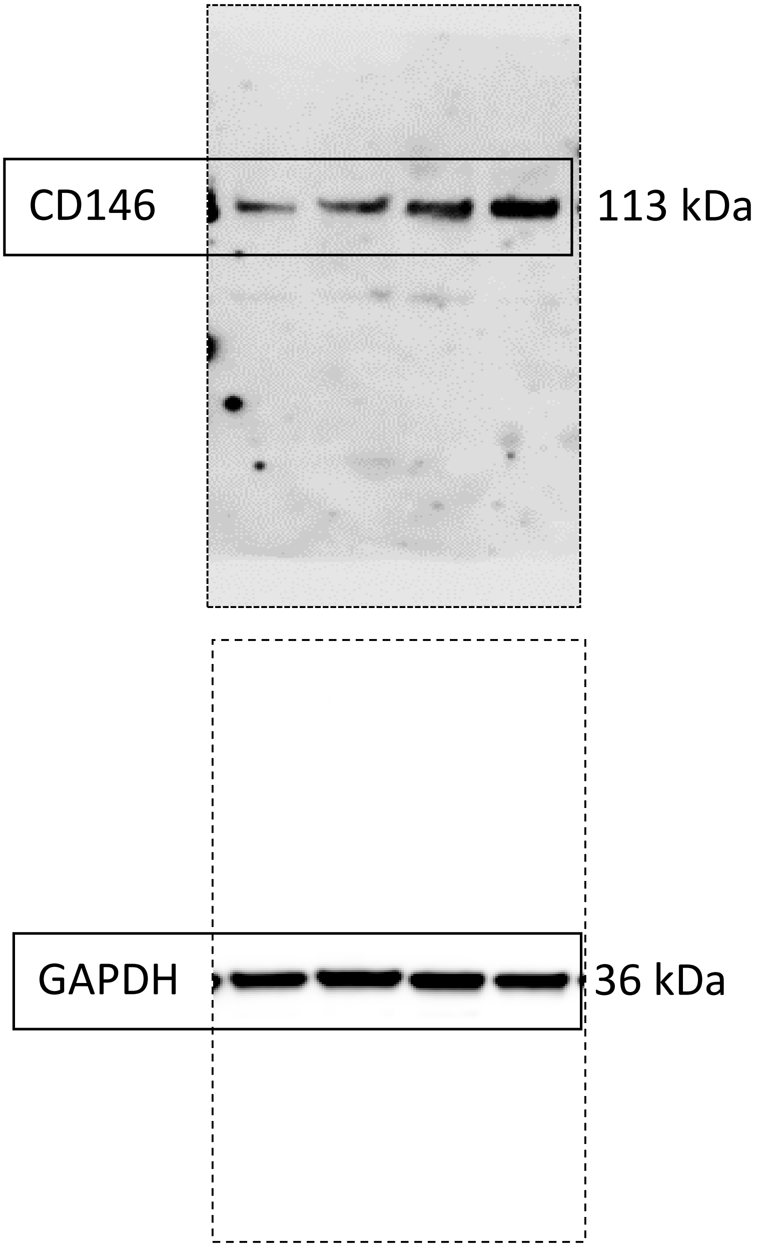

Figure 4B

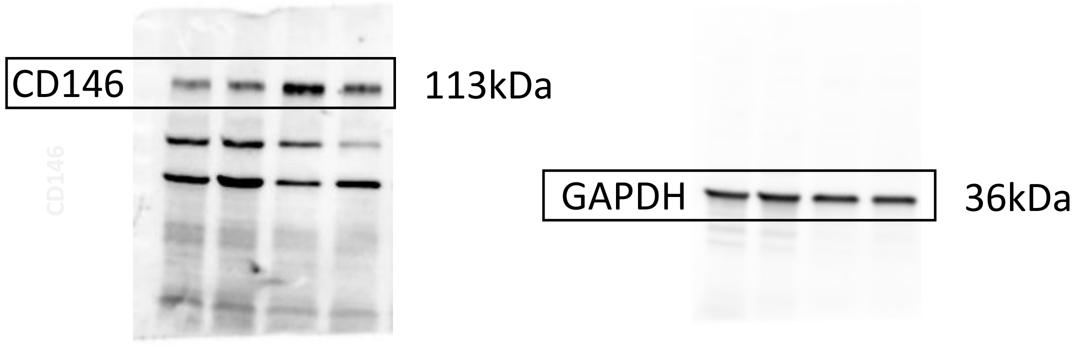

Figure 5A

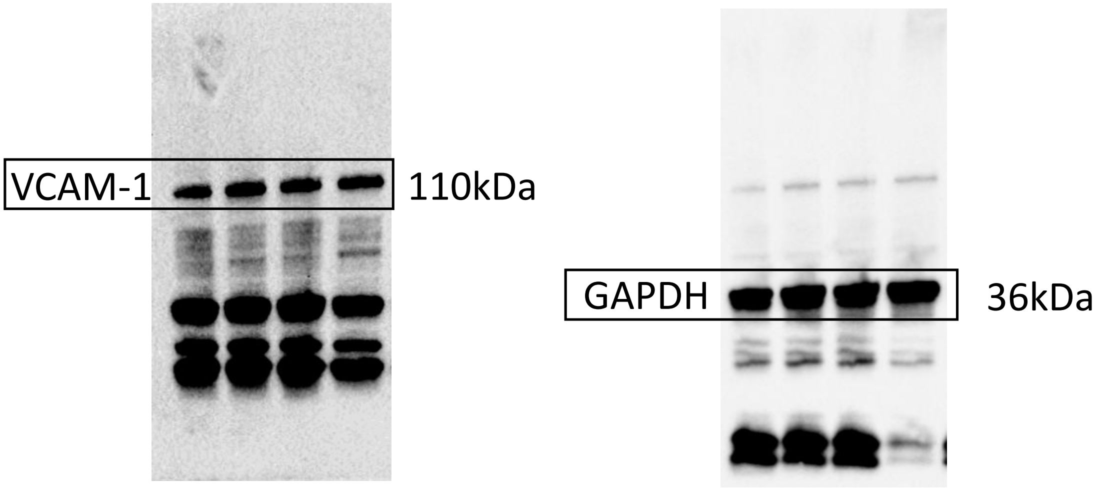

Figure 5E

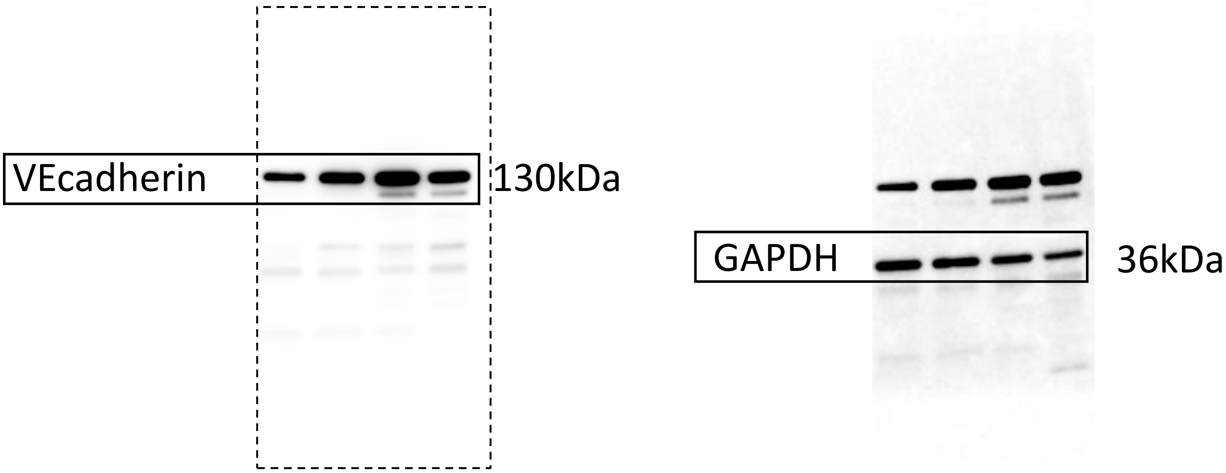

Figure 5G

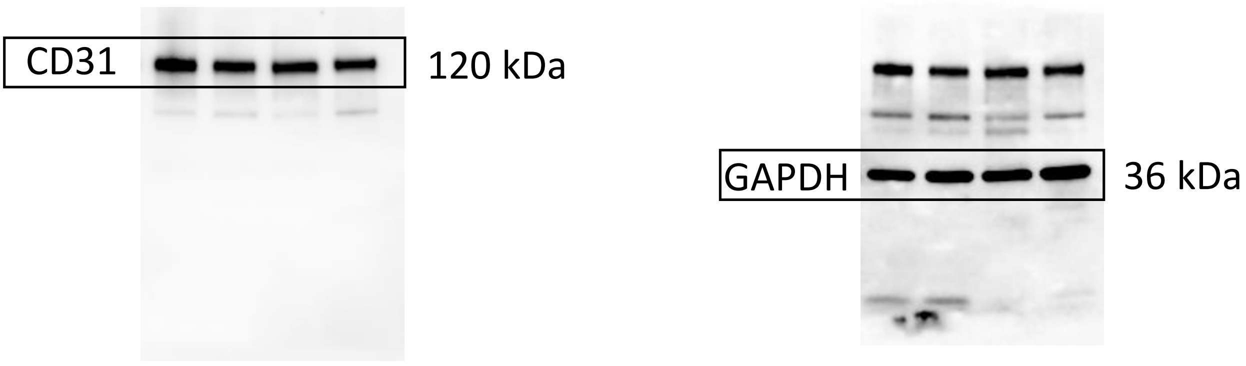

Figure 5I

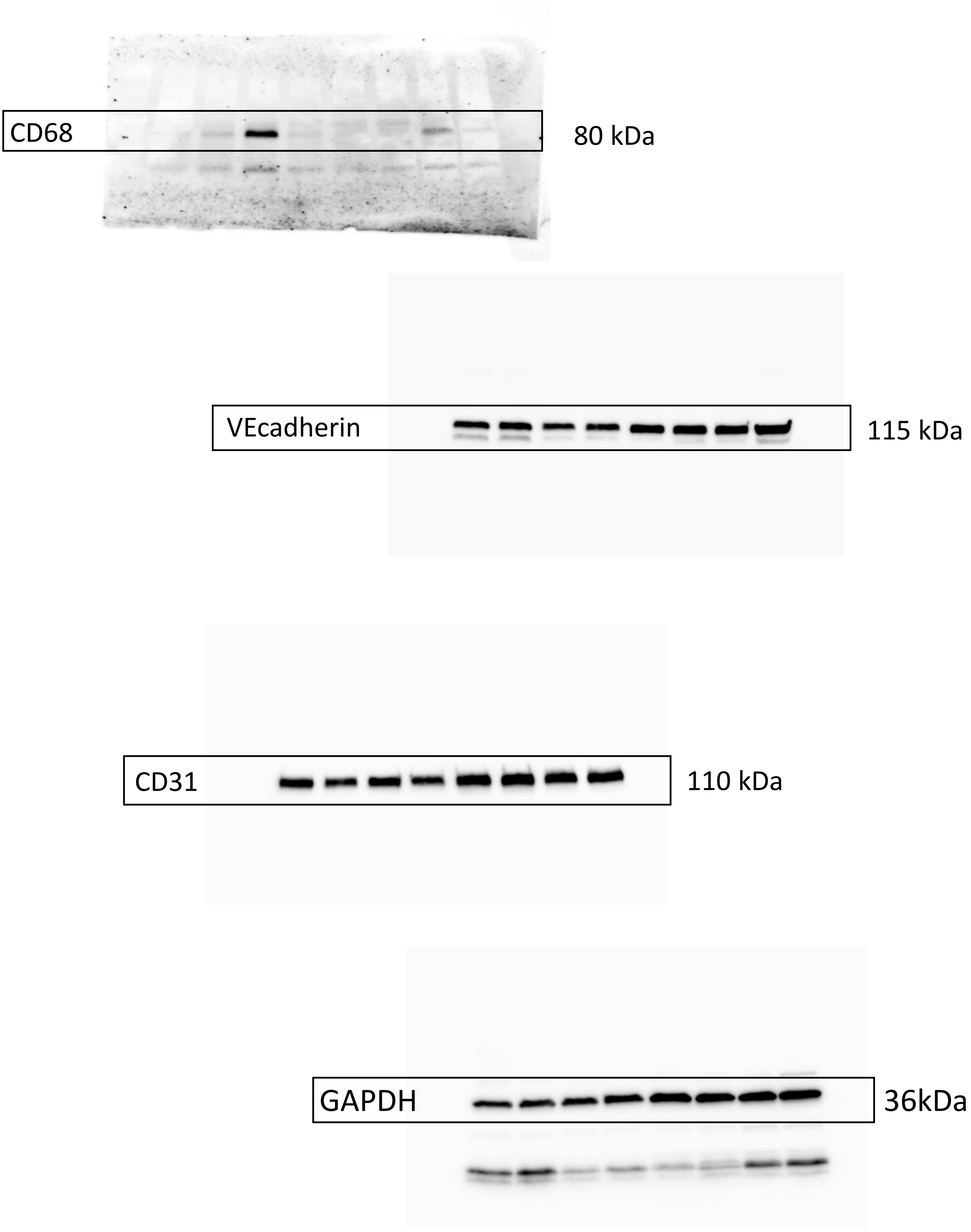

Figure 6I

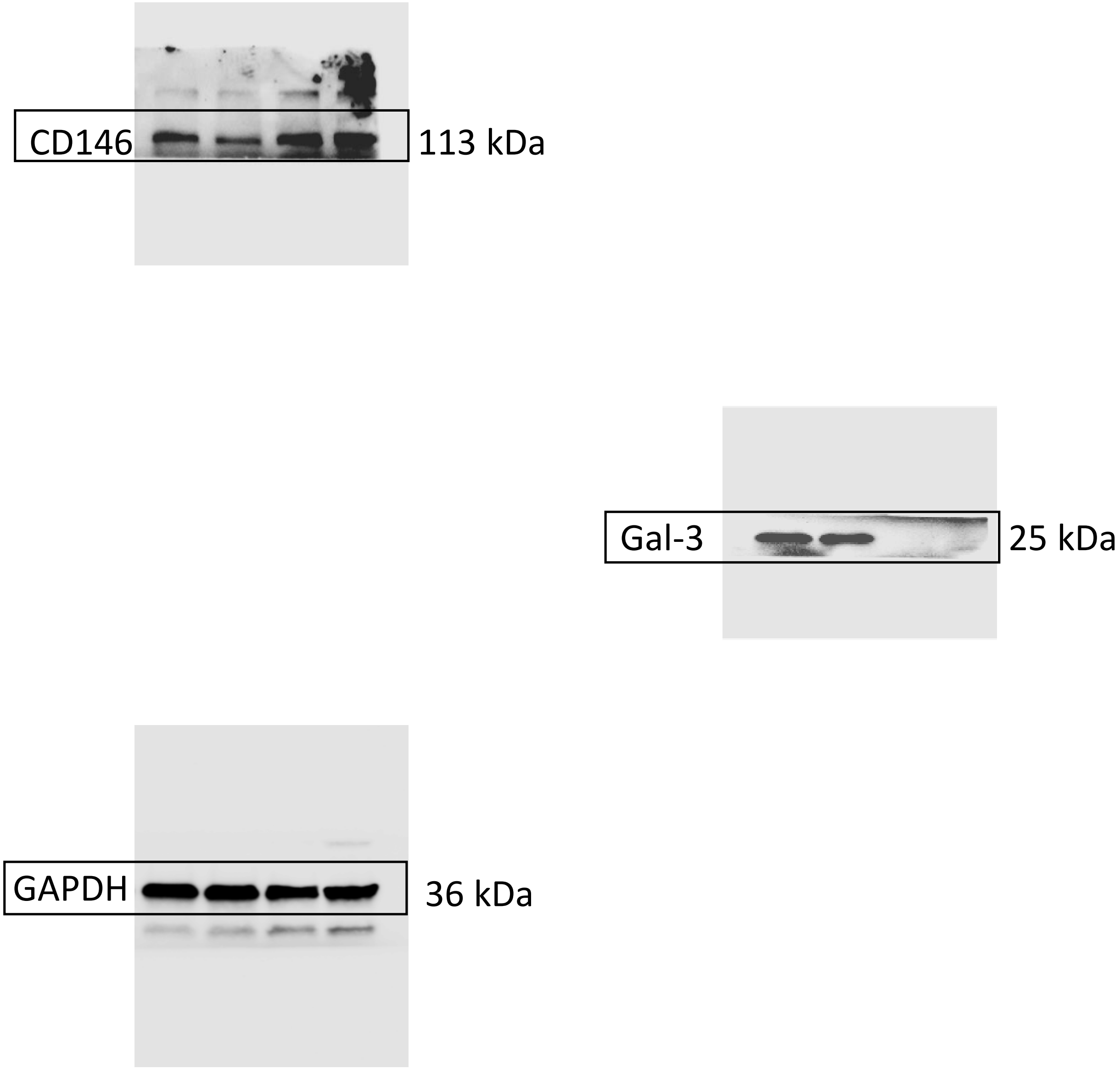

Figure 6J

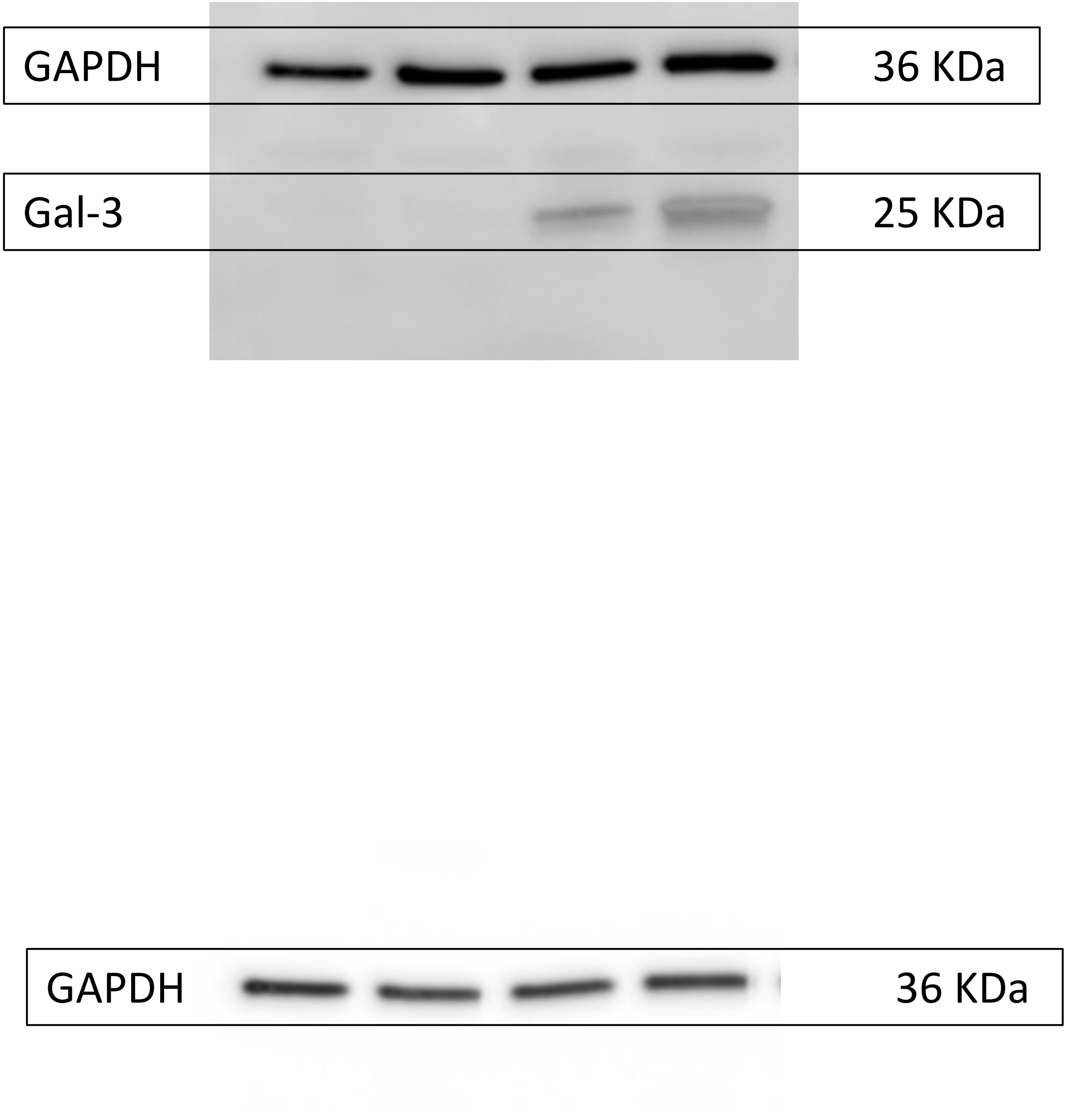

Supplementary Figure 5

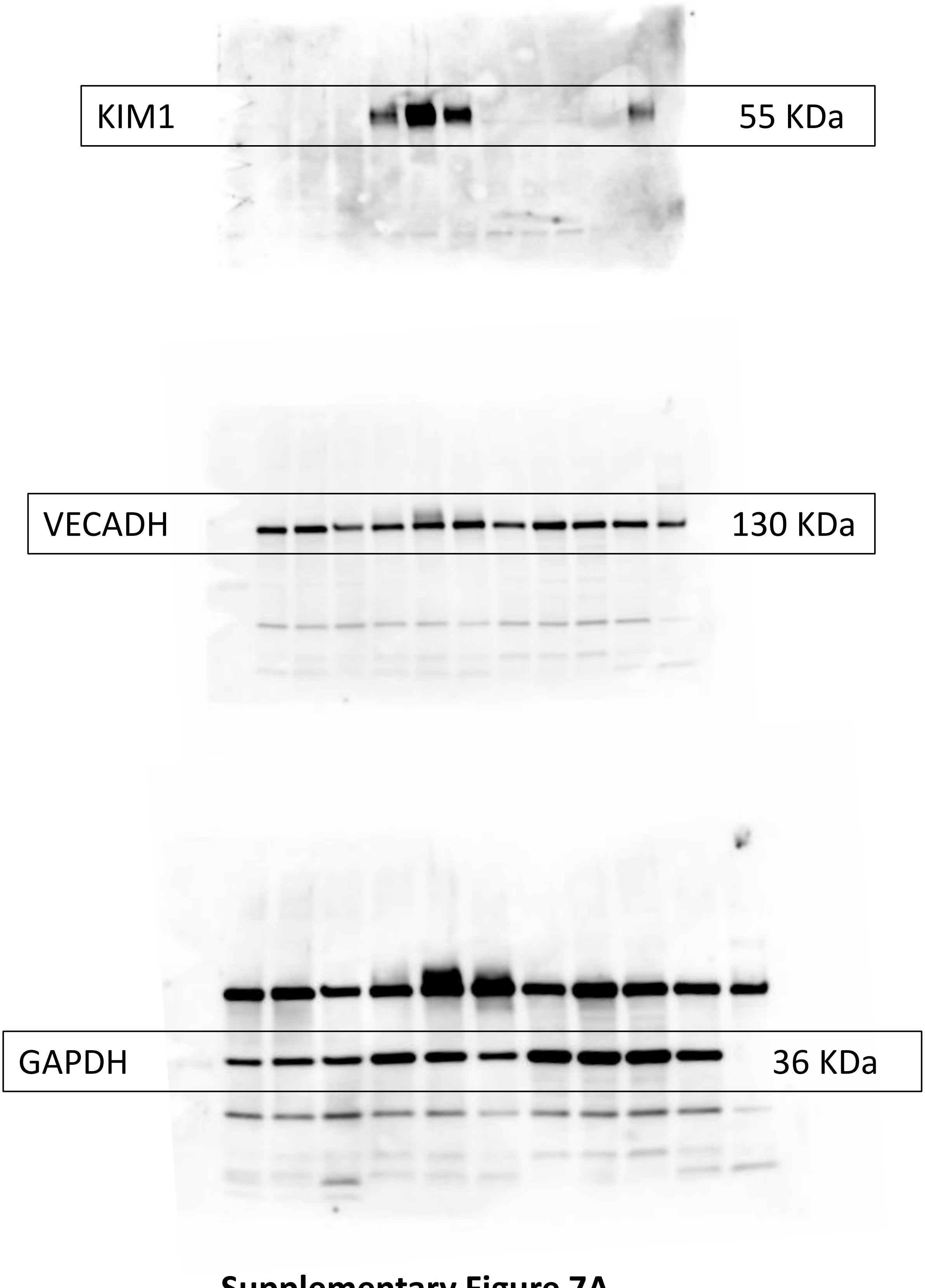

Supplementary Figure 7A

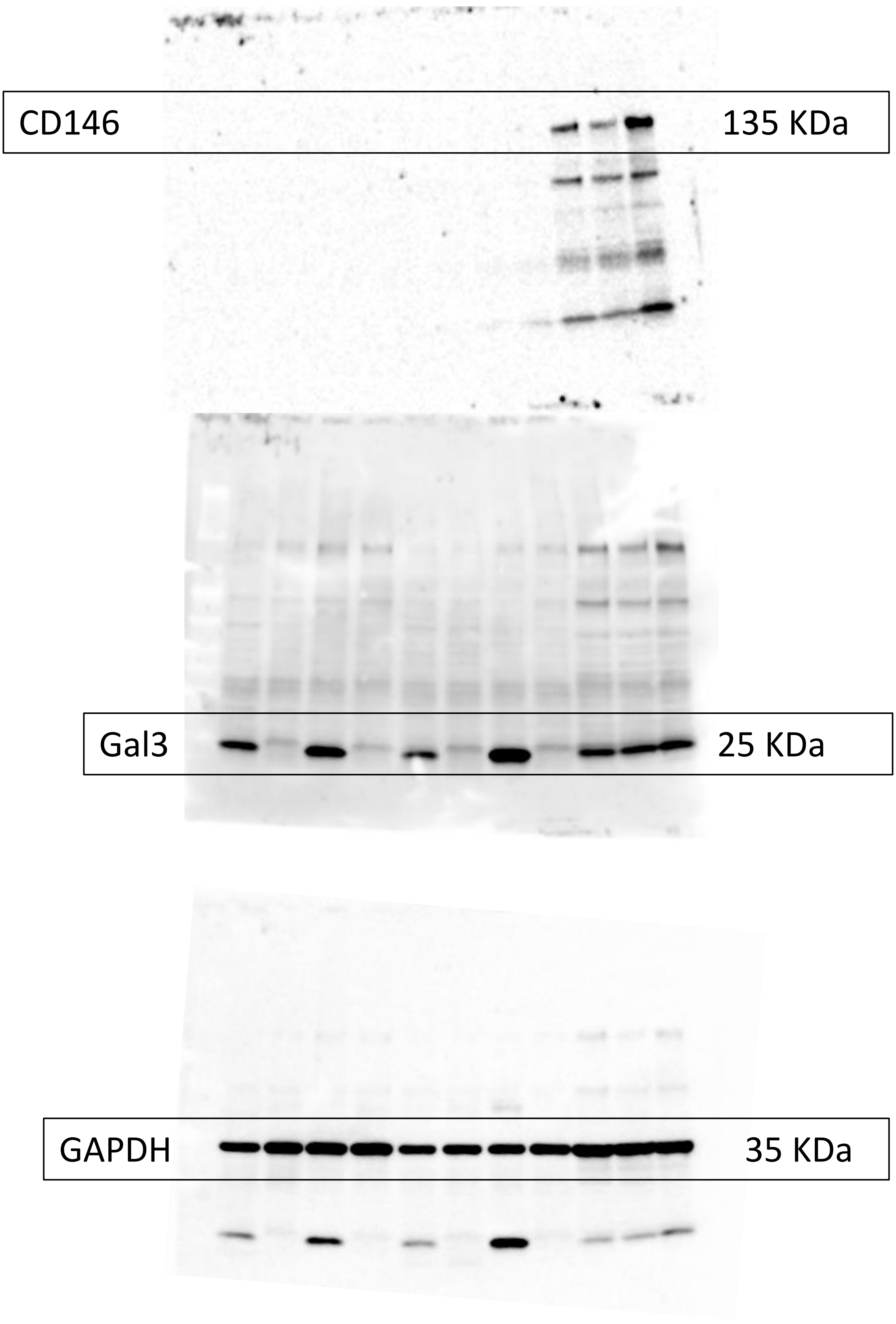

Supplementary Figure 7B

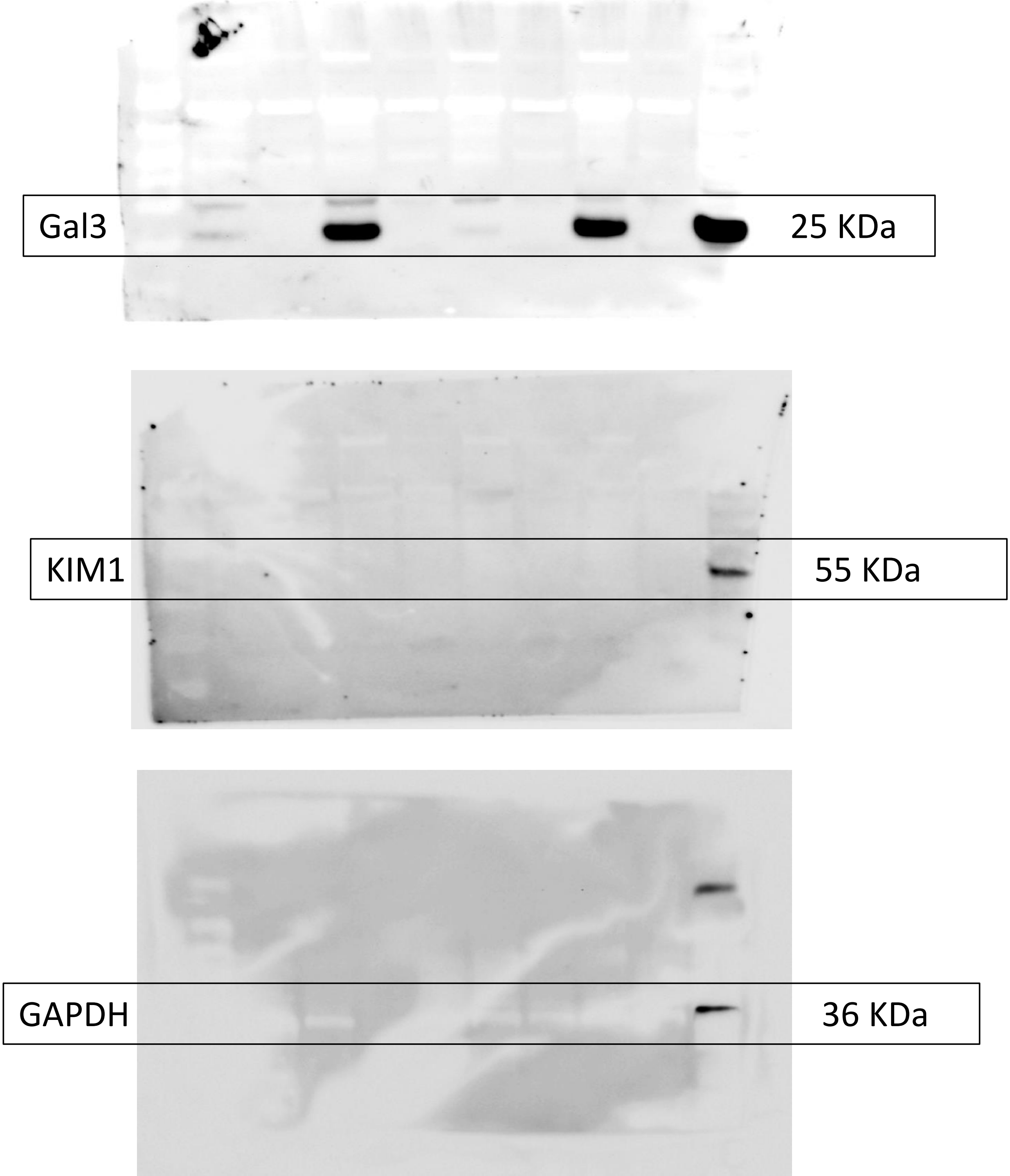

Supplementary Figure 8F

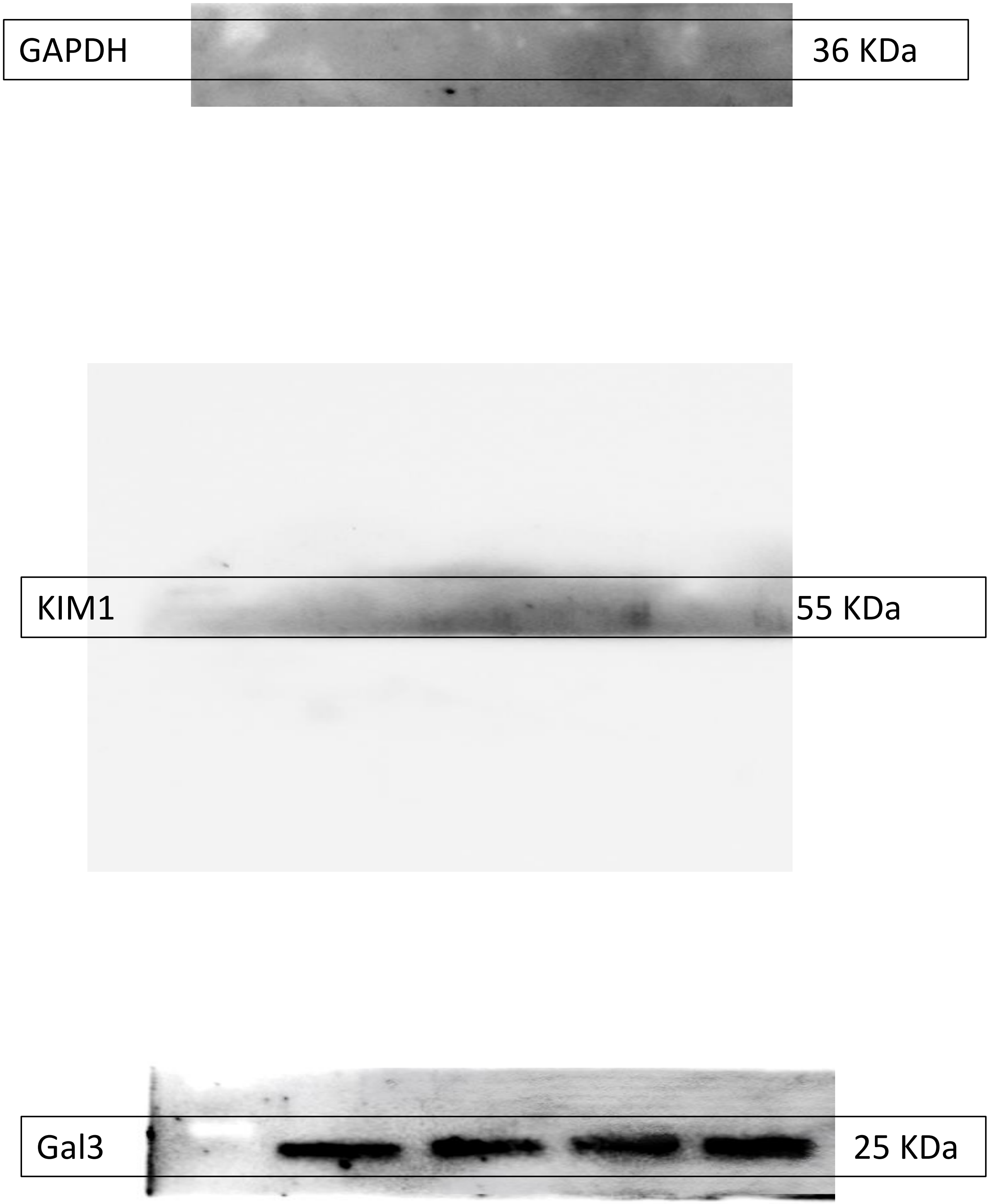

Supplementary Figure 8G
